# A gap-free reference genome reveals structural variations associated with flowering time in rapeseed (*Brassica napus*)

**DOI:** 10.1093/hr/uhad171

**Published:** 2023-08-29

**Authors:** Bao Li, Qian Yang, Lulu Yang, Xing Zhou, Lichao Deng, Liang Qu, Dengli Guo, Rongkui Hui, Yiming Guo, Xinhong Liu, Tonghua Wang, Lianyi Fan, Mei Li, Mingli Yan

**Affiliations:** Crop Research Institute, Hunan Academy of Agricultural Sciences, Changsha, Hunan 410125, China; Hunan Hybrid Rapeseed Engineering and Technology Research Center, Changsha, Hunan 410125, China; Crop Research Institute, Hunan Academy of Agricultural Sciences, Changsha, Hunan 410125, China; Hunan Hybrid Rapeseed Engineering and Technology Research Center, Changsha, Hunan 410125, China; Department of Cell Biology and Genetics, Wuhan Benagen Tech Solutions Company Limited, Wuhan, Hubei 430021, China; Crop Research Institute, Hunan Academy of Agricultural Sciences, Changsha, Hunan 410125, China; Hunan Hybrid Rapeseed Engineering and Technology Research Center, Changsha, Hunan 410125, China; Crop Research Institute, Hunan Academy of Agricultural Sciences, Changsha, Hunan 410125, China; Hunan Hybrid Rapeseed Engineering and Technology Research Center, Changsha, Hunan 410125, China; Crop Research Institute, Hunan Academy of Agricultural Sciences, Changsha, Hunan 410125, China; Hunan Hybrid Rapeseed Engineering and Technology Research Center, Changsha, Hunan 410125, China; Department of Cell Biology and Genetics, Wuhan Benagen Tech Solutions Company Limited, Wuhan, Hubei 430021, China; Crop Research Institute, Hunan Academy of Agricultural Sciences, Changsha, Hunan 410125, China; Hunan Hybrid Rapeseed Engineering and Technology Research Center, Changsha, Hunan 410125, China; Crop Research Institute, Hunan Academy of Agricultural Sciences, Changsha, Hunan 410125, China; Hunan Hybrid Rapeseed Engineering and Technology Research Center, Changsha, Hunan 410125, China; Crop Research Institute, Hunan Academy of Agricultural Sciences, Changsha, Hunan 410125, China; Hunan Hybrid Rapeseed Engineering and Technology Research Center, Changsha, Hunan 410125, China; Crop Research Institute, Hunan Academy of Agricultural Sciences, Changsha, Hunan 410125, China; Hunan Hybrid Rapeseed Engineering and Technology Research Center, Changsha, Hunan 410125, China; Crop Research Institute, Hunan Academy of Agricultural Sciences, Changsha, Hunan 410125, China; Hunan Hybrid Rapeseed Engineering and Technology Research Center, Changsha, Hunan 410125, China; Crop Research Institute, Hunan Academy of Agricultural Sciences, Changsha, Hunan 410125, China; Hunan Hybrid Rapeseed Engineering and Technology Research Center, Changsha, Hunan 410125, China; Crop Research Institute, Hunan Academy of Agricultural Sciences, Changsha, Hunan 410125, China; Hunan Hybrid Rapeseed Engineering and Technology Research Center, Changsha, Hunan 410125, China

## Abstract

Allopolyploid oilseed rape (*Brassica napus*) is an important oil crop and vegetable. However, the latest version of its reference genome, with collapsed duplications, gaps, and other issues, prevents comprehensive genomic analysis. Herein, we report a gap-free assembly of the rapeseed cv. Xiang5A genome using a combination of ONT (Oxford Nanopore Technologies) ultra-long reads, PacBio high-fidelity reads, and Hi-C datasets. It includes gap-free assemblies of all 19 chromosomes and telomere-to-telomere assemblies of eight chromosomes. Compared with previously published genomes of *B. napus*, our gap-free genome, with a contig N50 length of 50.70 Mb, has complete assemblies of 9 of 19 chromosomes without manual intervention, and greatly improves contiguity and completeness, thereby representing the highest quality genome assembly to date. Our results revealed that *B. napus* Xiang5A underwent nearly complete triplication and allotetraploidy relative to *Arabidopsis thaliana*. Using the gap-free assembly, we found that 917 flowering-related genes were affected by structural variation, including *BnaA03.VERNALIZATION INSENSITIVE 3* and *BnaC04.HIGH EXPRESSION OF OSMOTICALLY RESPONSIVE GENES 1*. These genes may play crucial roles in regulating flowering time and facilitating the adaptation of Xiang5A in the Yangtze River Basin of China. This reference genome provides a valuable genetic resource for rapeseed functional genomic studies and breeding.

## Introduction

*Brassica napus* (AACC, 2*n* = 38) is a member of Brassicaceae, an important family that includes numerous oil and vegetable crops with high agricultural significance [1]. Rapeseed is a globally distributed oilseed crop, accounting for >13% of edible oil production worldwide (https://www.ers.usda.gov/). Furthermore, its sprouts, shoots, and leaves are marketed as functional nutritious vegetables in China because of their high levels of basic and essential nutrients [2, 3]. In addition, the role of rapeseed flowers in promoting the ornamental tourism industry has recently gained considerable attention in China [4, 5]. Therefore, rapeseed possesses economic value not only as oilseed, but also holds nutritional and aesthetic values, making it suitable for utilization as both a vegetable and an ornamental plant in horticulture.

Oilseed rape arose from allopolyploidy between *Brassica rapa* and *Brassica oleracea* [6]. Owing to duplications and polyploidization, its genome is extremely complex and difficult to resolve [1, 6–8]. The first rapeseed reference genome, Darmor-*bzh* v4.0, was released in 2014 using Illumina short reads [6]. Several reference genomes have recently been established based on Illumina short reads, Oxford Nanopore Technologies (ONT) reads, high-error-rate PacBio reads, and chromosome conformation capture (Hi-C) data [9–11]. For example, using PacBio and Hi-C data, the genome ZS11_HZAU was assembled [11]. These assemblies have been used for gene mapping, functional analysis, and genomic research. Although some genomes have been constructed, rapeseed reference genomes still suffer from several issues that hinder their application. In the latest ZS11_HZAU genome, more than 50 million nucleotides remain completely unresolved, and the telomeres are not mentioned [11]. The quality of the Darmor-*bzh* v10 genome has been significantly enhanced in its upgraded version, achieved through the use of ONT and Illumina reads, supplemented by optical and genetic maps, resulting in a contig N50 that has now reached 11.49 Mb [10]. However, the number of unanchored sequences still exceeds 6%, and there is no mention of telomere-to-telomere (T2T) assembly of any chromosome [9–11]. These unresolved sequences or gaps almost certainly contain important functional genes [12]. Overall, these issues introduce biases in functional genetic and genomic analyses.

Recent improvements in sequencing technologies and assembly algorithms have facilitated the generation of more complete assemblies of complex genomes [13–17]. PacBio high fidelity (HiFi) provides a compromise between long reads (10–20 kb) and low error rates (< 0.1%) [18]. ONT provides ultra-long (> 100 kb) reads, filling the gaps present in the HiFi-assembled reference [14]. Several gap-free assemblies combining these two methods have been reported [17, 19–24]. For example, integrated HiFi and ONT ultra-long reads, resulting in four gap-free genomes for rice, were recently reported. A comparison of the HiFi and ONT assemblies showed that the former provides high accuracy, while the latter provides high continuity [25]. Using this method, the first complete human genome, T2T-CHM13, was produced, and is complete and more accurate when compared with previous genomes, with almost all structural errors in its predecessors corrected. It also added nearly 200 Mb of sequence, filling 120.31 Mb of gaps [20, 21]. In recent years, based on the integration of HiFi and ONT long reads, several plant T2T genomes have been assembled, including kiwifruit [22] and grapevine [26]. These findings show that combining these two methods is currently the most efficient way to assemble complex genomes [17, 25].

Using gap-free genomes offers an unprecedented opportunity for more comprehensive genome annotation and a global view of the architecture of repeat elements, centromeres, and duplicated genes. They facilitate evolutionary genomic studies and more comprehensive identification of structural variations (SVs) and presence/absence variations (PAVs) [20, 24, 27]. SVs and PAVs are important in phenotypes, disease, evolution, and agriculture; however, SV discovery using incomplete reference genomes is extremely difficult and unreliable. Gap-free genomes significantly reduce false SVs and PAVs and improve the balance of the numbers of insertions and deletions. Additionally, they enable the detection of *de novo* and additional SVs within previously uncharacterized sequences, offering a broader view of genome-wide SVs and PAVs [20, 28].

SVs have been identified as the causative variations underlying several important traits in rapeseed, including the flowering time and ecotype (winter, semi-winter, and spring) differentiation [11, 28]. Several significant SVs were identified in the *BnaA10.FLOWERING LOCUS C* (*FLC*) and *BnaA02.FLC* genes, including a 4.422-kb hAT, a 621-bp miniature inverted-repeat transposable element (MITE), a 5.625-kb long interspersed nuclear element transposon insertion in *BnaA0.FLC*, and an 810-bp miniature MITE in *BnaA02.FLC*. The presence of the 621-bp MITE insertion in *BnaA10.FLC*, along with a functional *BnaA02.FLC*, seems to be a crucial combination for the development of the winter/semi-winter ecotypes of rapeseed [29]. In addition, several genes have been implicated in the regulation of flowering time; however, the causative variations associated with them, including *BnaA03.VERNALIZATION INSENSITIVE 3* (*VIN3*), have not been identified [30].

Gap-free assembly is the beginning of a new era that will allow the comprehensive exploration of genomic variation and functional genes. Since a gap-free genome was lacking in rapeseed, we report in this study the first rapeseed gap-free assembly of cv. Xiang5A obtained by integrating HiFi, ONT long, and Hi-C sequencing data. Our genome will greatly help future genomic research and uncover previously missed functional genes and genomic variants.

## Results

### Genome assembly

**Figure 1:**
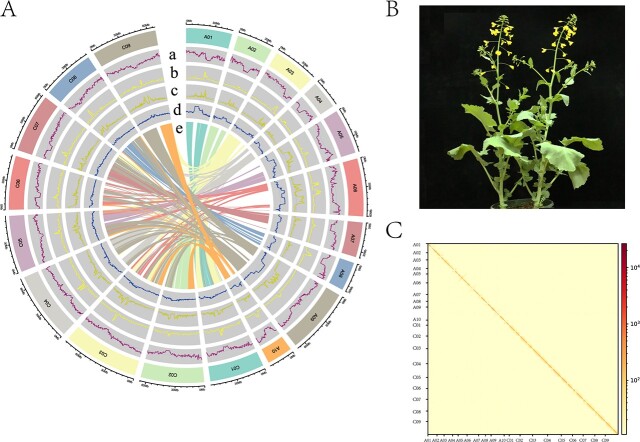
Overview of the ‘Xiang5A’ (X5A) genome and phenotype. (**A**) Circos plot. From the outer to inner ring: (a) gene number density, (b) LTR/Copia density, (c) LTR/Gypsy density, (d) GC content, and (e) syntenic block. (**B**) Phenotype. Scale bar = 5 cm. (**C**) Hi-C chromatin interaction map of the X5A genome. Color intensities indicate frequencies of interaction between pairs of loci, each spanning 500 kb.

Xiang5A (X5A), a shared paternal line of several elite hybrid varieties, including FengYou737 (FY737), was chosen for sequencing (Fig. 1B). FY737 is a hybrid variety bred by the Hunan Crop Research Institute, known for its early maturity, high yield, and adaptability. Using *k*-mer analysis of Illumina reads, we assessed the size of the X5A genome and obtained a *k*-mer value of 19. Based on this, we estimated the genome size as 1033.31 Mb, with a heterozygosity level of 0.22% (Supplementary Data Fig. S1; Supplementary Data Table S1). To assemble the genome, we performed extensive sequencing using multiple technologies, including 66.69 Gb (64.75× coverage of the estimated 1033 Mb nuclear genome) PacBio HiFi reads, 66.19 Gb (64.26× coverage) ONT ultra-long reads, and 131.80 Gb (129.04× coverage) Illumina Arima Genomics Hi-C sequence (Supplementary Data Table S2). The read length N50 values were 100.29 and 15.86 kb for ONT and PacBio reads, respectively (Supplementary Data Table S2). The ONT reads were independently assembled using the bioinformatics tools NextDenovo, Flye, and NECAT, yielding contig N50 values of 31, 16, and 13 Mb, respectively (Supplementary Data Table S3). ONT and HiFi reads were integrated using hifiasm, yielding 651 contigs, with a combined size of 1045 Mb and an N50 length of 50.70 Mb; they were 4.4-, 31-, 34-, and 1267-fold longer than earlier released genome versions (Darmor-*bzh* v10, ZS11_PB, ZS11_HZAU, and Darmor-*bzh* v4.1, respectively; Table 1; Supplementary Data Table S3). The hifiasm assembly consisted of contiguous sequences that spanned the entire length of chromosomes A03, A05, A07, A08, A10, C02, C07, C08, and C09 (Supplementary Data Tables S4 and S5). The completeness of these assemblies exceeded 99% when evaluated by Benchmarking Universal Single-Copy Orthologs (BUSCO) analysis (Supplementary Data Table S3).

**Table 1:** Assembly statistics of rapeseed genomes.

**Assembly name**	**X5A**	**Darmor-*bzh* v10**	**ZS11_HZAU**	**ZS11_PB**	**ZS11_NGS**	**Darmor-*bzh* v4.1**
Principal sequencing technology	ONT; HiFi; Hi-C	ONT	PacBio; Hi-C	PacBio	NGS	454
Total size of the assembled sequence (Mb)	1045	924	1008	921	976	850
Anchored chromosome (Mb)	1005	867	961	858	855	645
Sequence was anchored to chromosomes (%)	96	94	95	0.93	0.88	0.76
Number of contigs	19	505	8773	4035	46 007	44 837
Contig N50 (Mb)	50.70	11.47	1.51	1.64	0.04	0.04
Number of scaffolds	19	237	3332		3460	
Gaps	0	268	5460			
Number of telomeres	25	0	0	0	0	0
Number of annotated genes	124 744	108 190	100 919	106 059	101 942	101 040
Average gene length (bp)	2492.8		2061	2731.1	2086.7	1953
Total size of TEs (Mb)	596.8	497.66	574.70	569.75	449.79	342.34
BUSCOs (%)	99.20	98.60	98.50	98.30	98.00	97.60
References		[10]	[11]	[9]	[31]	[6]

Owing to the high accuracy of PacBio HiFi reads, we used the hifiasm initial assembly as backbone. These sequences were then clustered, ordered, and oriented using the Hi-C data. A total of 1 006 846 396 bp of hifiasm assembly sequence was anchored to 19 pseudochromosomes, with 13 gaps distributed across 10 of the pseudochromosomes (Supplementary Data Tables S4 and S5). The ONT and HiFi contigs, along with the ONT and HiFi raw reads, were used to fill gaps, all of which were filled with bridged contigs or raw reads (Supplementary Data Fig. S2). Finally, a gap-free genome was assembled, consisting of 19 gap-free chromosomes with a total length of 1004.95 Mb (Table 1; Fig. 1A).

### Genome annotation

The near-complete assembly of X5A provides an unprecedented opportunity to comprehensively annotate 580.09 Mb of repeating sequences (57.72% of the genome) predicted using a repeat sequence library and RepBase (Table 2). Using RepeatProteinMask, we identified 135 651 229 bp of transposable element (TE) proteins, which accounted for 13.50% of the assembly. Finally, after removing redundant portions of repeats, 596 802 348 bp of repetitive sequences was identified, representing 59.39% of the genome. Among them, long terminal repeat (LTR) retrotransposable elements represent the largest class, accounting for 271.23 Mb (26.99% of the genome) (Table 2).

**Table 2:** Summary statistics of repetitive sequences in X5A genome assembly.

	**TE proteins**		**De novo + RepBase**		**Combined TEs**	
**Type**	**Length (bp)**	**% in genome**	**Length (bp)**	**% in genome**	**Length (bp)**	**% in genome**
DNA	20.68	2.06	183.56	18.27	184.38	18.35
LINE	22.12	2.2	70.46	7.01	72.42	7.21
SINE	0.00	0	2.43	0.24	2.43	0.24
LTR	92.84	9.24	270.49	26.92	271.23	26.99
Satellite	0.00	0	47.71	4.75	47.71	4.75
Simple repeat	0.00	0	0.01	1.47E−03	0.01	1.47E−03
Other	0.00	0	0.00	1.20E−04	0.00	1.20E−04
Unknown	0.03	0	89.34	8.89	89.37	8.89
Total	135.65	13.5	580.09	57.72	596.80	59.39

**Figure 2:**
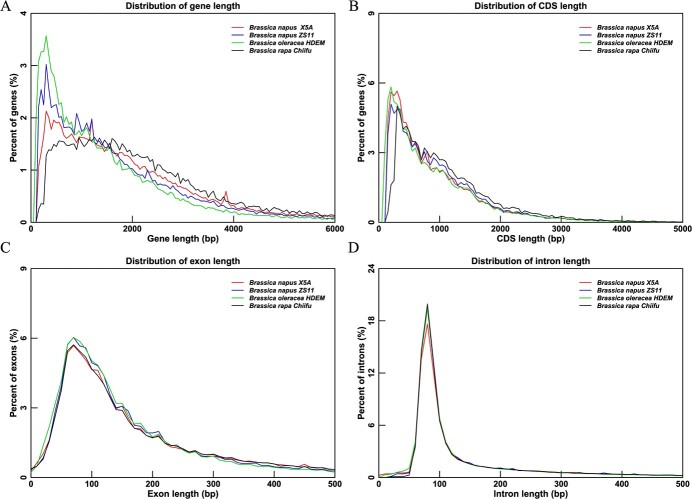
Distribution of the length of gene and gene components in the X5A assembly. (**A**) Gene length. **(B**) Coding sequence (CDS) length. (**C**) Exon length. (**D**) Intron length. The *y*-axis represents the percentage of genes, CDS, exons, and introns with a certain length.

We next utilized an automated annotation pipeline supported by Iso-Seq transcriptome data, *ab initio* prediction, and protein homology [32, 33]. Based on the improved completeness, X5A surpassed the previous assembly in gene number. In total, 124 774 gene models were identified in the X5A genome (Supplementary Data Table S6), capturing 99.2% of a BUSCO 1614 reference gene set. Our assembly contains ~24 000 more genes than previous assemblies (Table 1; Supplementary Data Table S7). The average gene length was 2493 bp, with an average 4.54 exons per gene (Fig. 2; Supplementary Data Table S6). Among the 124 774 genes, 121 229 (97.16%) were functionally annotated based at least on one type of functional database (Supplementary Data Table S8). Transcriptome data supported 54 722 genes, accounting for 43.86% of the total (Supplementary Data Tables S9 and S10). In addition, 21 867 (5.79 Mb) non-coding RNAs were predicted, representing 0.57% of the genome, including 479 microRNAs, 2433 transfer RNAs, 14 983 ribosomal RNAs, and 3981 small nuclear RNAs (Supplementary Data Table S11).

### Centromere analyses

**Figure 3:**
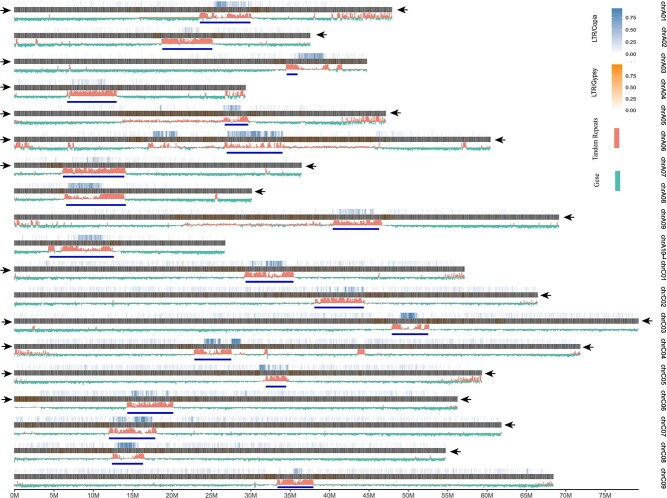
Delimiting of X5A centromeres. Layers of each chromosome graph (top to bottom) indicate (1) LTR/Copia distribution, (2) LTR/Gypsy distribution, (3) tandem repeat distribution, and (4) non-TE gene distribution. Arrows represent telomeric sequence repeats and boxes beneath the chromosome represent the predicted centromere locations.

Centromeres play a crucial role in maintaining chromosome stability and ensuring accurate chromosome segregation during cell division. The Tandem Repeats Finder tool was used to identify putative centromeres and their boundaries (Fig. 3; Supplementary Data Table S12). Putative centromeres varied in size from 1.30 to 8.07 Mb; most were longer than those in the ZS11_HZAU assembly except for those of chromosomes A03, A05, and C04 (Supplementary Data Table S12). Abundant TEs were found, comprising 98.49% of centromeres, of which LTRs accounted for >35% of repetitive sequences (Supplementary Data Table S13).

### Assessment of X5A genome assembly

BUSCO was first used to assess completeness. Over 99.0% (1598/1614) of the reference gene set was complete in the X5A assembly (Supplementary Data Table S7). Second, we mapped HiFi and ONT reads to the X5A draft assembly; the centromeric regions exhibited lower-than-average HiFi coverage (Supplementary Data Fig. S3; Supplementary Data Table S14), possibly because of the increased annealing of CT/GA-rich sequences during HiFi library preparation, rather than assembly error. Similar observations have been reported for the tomato and human genomes [21, 34]. All ONT raw sequences evenly covered the 19 chromosomes, including centromeres and gap regions (Supplementary Data Fig. S4). We observed that 99.95, 99.99, and 100% of the Illumina, HiFi, and ONT reads, respectively, aligned successfully to the X5A assembly. All three types of reads covered 100% of the loci in the assembly, indicating high accuracy and completeness (Supplementary Data Table S14). Third, we evaluated draft assembly quality using *k*-mer analysis, obtaining a quality value (QV) of 58.09 for the entire assembly. The 19 chromosomes exhibited high accuracy, with QVs ranging from 50.07 to 63.00 (Supplementary Data Table S15). Fourth, to confirm genome structure, we aligned paired Hi-C reads to the genome and analyzed the resulting contact map, which provided robust support for the 19 well-assembled chromosomes and revealed no apparent misjoins (Fig. 1C). Subsequently, repeat sequence annotation was performed, revealing an LTR assembly index (LAI) value of 20.03, meeting the criterion of a high-quality genome. Finally, 25 telomeres were identified using the telomeric repeat (CCCTAAA/TTTAGGG) as a query, resulting in eight T2T pseudomolecules for the entire genome (Supplementary Data Table S16; Fig. 3). Overall, these results confirmed the accuracy and completeness of the assembly.

### Global comparisons with previous genomes

**Figure 4:**
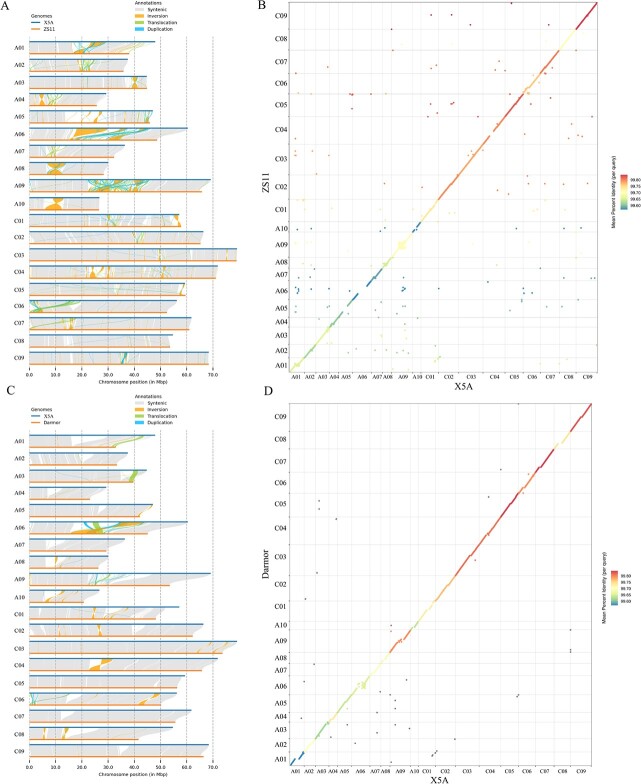
Collinearity between X5A and previous rapeseed genome assemblies. (**A**) Structural variations between the X5A and ZS11_HZAU genomes, with X5A as the reference. (**B**) Collinearity of the genomes of *B. napus* X5A and ZS11_HZAU. (**C**) Structural variations between the X5A and Darmor-*bzh* v10 genomes. (**D**) Collinearity of the genomes of *B. napus* X5A and Darmor-*bzh* v10.

The contiguity and completeness of our gap-free X5A genome were both higher than those of previously reported assemblies; the contig N50 was 50.70 Mb, far exceeding those of all other assemblies (Table 1). More importantly, chromosomes A03, A05, A07, A08, A10, C02, C07, C08, and C09 were completely assembled gap-free, without manual intervention (Supplementary Data Table S5). Previous reference genomes contain hundreds or thousands of gaps; however, all gaps were filled in our assembly. Our assembly was longer than all previous assemblies. All chromosomes in the X5A assembly were longer than those in the Darmor-*bzh* v10, ZS11_PB, and Darmor-*bzh* v4.1 assemblies, while all 17 other X5A pseudochromosomes were longer than those in ZS11_HZAU, except chromosomes C01 and C05 (Fig. 4; Supplementary Data Table S17). The X5A assembly includes 25 telomeres, whereas no telomere was identified in the previous genomes (Fig. 3; Supplementary Data Table S16). Our assembly had a BUSCO value >99.0%, higher than those of previously published assemblies (Table 1; Supplementary Data Table S7). In conclusion, X5A is the highest-quality rapeseed reference genome to date.

The X5A assembly adds >50 Mb that is not collinear with the ZS11_HZAU genome [11], which likely contains important functional genes. In total, 2706 newly annotated genes were identified in these regions; 2441 (90.2%) were functionally annotated using at least one type of functional database (Supplementary Data Table S18). To determine the functions of the newly annotated genes, we conducted Gene Ontology (GO) enrichment analysis, revealing significant enrichment of genes associated with the regulation of protein localization to the cell surface, regulation of double fertilization forming a zygote and endosperm, ADP binding, and vesicle (Supplementary Data Fig. S5). Several newly annotated genes function in phytohormone signaling and biosynthesis, including the auxin-responsive factors *auxin response factor 18* and *SMALL AUXIN UPREGULATED RNA 46.* There were also several transcription factors, including those encoding homeobox protein 20 and WRKY DNA-binding protein 33 (Supplementary Data Table S18). These proteins may have significant roles in the growth and development of rapeseed.

**Figure 5:**
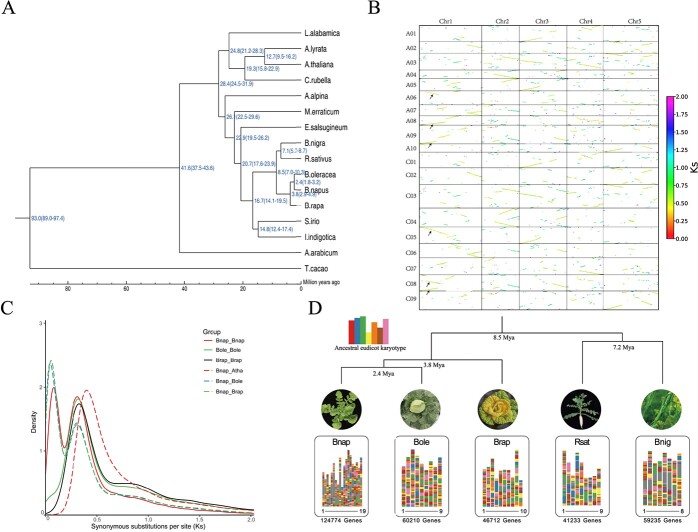
Evolutionary history of the *B. napus* genome. (**A**) Phylogenetic tree with the estimated divergence times of *Theobroma cacao* and 15 Brassicaceae genomes. *A. thaliana*, *Arabidopsis thaliana*; *A. alpina*, *Arabis alpina*; *A. arabicum*, *Arabidopsis arabicum*; *A. lyrata*, *Arabidopsis lyrata*; *B. oleracea*, *Brassica oleracea*; *B. nigra*, *Brassica nigra*; *B. napus*, *Brassica napus*; *B. rapa*, *Brassica rapa*; *C. rubella*, *Capsella rubella*; *E. salsugineum*, *Eutrema salsugineum*; *I. indigotica*, *Indigofera indigotica*; *L. alabamica*, *Leavenworthia alabamica*; *M. erraticum*, *Mimulus erraticus*; *R. sativus*, *Raphanus sativus*; *S. irio*, *Sisymbrium irio*. (**B**) Collinearity of *B. napus* X5A and *A. thaliana* genomes. (**C**) Frequency distributions of synonymous substitution rates (*K*_s_) between collinear genes in syntenic blocks. The synonymous replacement rate was 2 × 1.4 × 10^−8^ mutations site/year. Atha, *A. thaliana*. (**D**) Synteny by comparisons with ancestral eudicot karyotype chromosomes; *K*_s_ values are shown as multicolored columns. Bnap, *B. napus*; Bole, *B. oleracea*; Brap, *B. rapa*; Bnig, *B. nigra*; Rsat, *R. sativus*.

### X5A genome evolution

**Figure 6:**
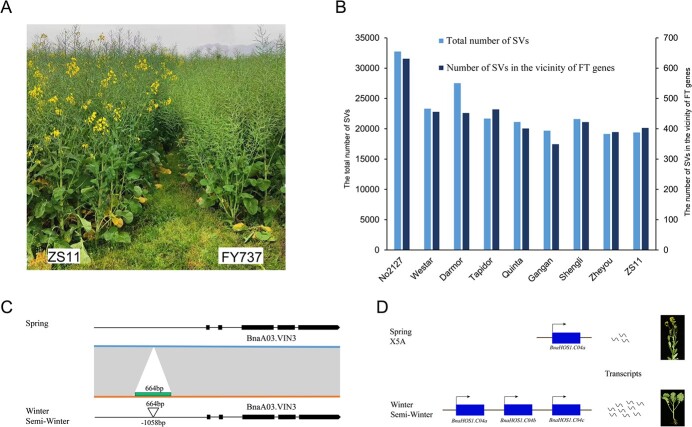
Structural variations in flowering-related genes. (**A**) Plant morphologies of ZS11 and FY737. FY737 is a hybrid variety with X5A as the maternal parent. (**B**) Total SVs and SVs associated with flowering time-related (FT) genes identified in X5A and nine rapeseed genomes. (**C**) Schematic of *BnaVIN3.A03*, with semi-winter/winter rapeseed (Darmor, Tapidor, Quinta Winter, ZS11, Gangan, Zheyou7, Shengli, and X5A) containing a 664-bp transposon insertion in the promoter. (**D**) Schematic of *BnaC04.HOS1*, with Darmor, Tapidor, Quinta, ZS11, Gangan, Zheyou7, and Shengli having a 49-kb tandem duplication containing two identical copies of *BnaC04.HOS1*.

We gathered genome sequences from 15 species for comparative genomic analysis with X5A to investigate its evolutionary history. OrthoFinder was utilized to cluster annotated genes. On average, there were 2.66 genes per family, with 124 774 genes classified into 46 851 gene families in the X5A genome (Supplementary Data Table S19). We employed the PROTGAMMAWAG model in RAxML for phylogenetic analysis using *Theobroma cacao* as the outgroup, yielding a Brassicaceae phylogeny for the 15 genomes (Fig. 5A), clearly indicating that the *B. oleracea* ancestral genomes were more closely related to X5A, as the divergence of *Brassica* from *Isatis indigotica* occurred ~16.7 million years ago (Mya; Fig. 5A). To study chromosome rearrangements within and among *Brassica* species, we reconstructed the ancestral eudicot karyotype (AEK). The identification of significant interchromosomal rearrangements in *Brassica* genomes suggests that large-scale whole-genome rearrangements and duplications were common (Fig. 5D). Apart from the region close to the centromere, there is a clear similarity in karyotype between the A01–A10 chromosomes of *B. napus* and the corresponding A01–A10 chromosomes of *B. rapa*. Likewise, the C01–C09 chromosomes of *B. napus* exhibit a similar karyotype to the matching C01–C09 chromosomes of *B. oleracea*. This suggests that the *B. napus* genome originated through the allotetraploidization of its diploid ancestors, *B. rapa* and *B. oleracea* (Fig. 5D). The karyotype of *B. oleracea* was most similar to that of *B. napus* X5A, in agreement with the phylogenetic analysis (Fig. 5A and D). Syntenic blocks were identified between X5A and the other *Brassica* genomes, and the *K*_s_ values of gene pairs within these blocks were estimated (Fig. 6C). The findings suggest that a whole-genome triplication occurred at ~11 Mya (*K*_s_ peak value ~0.30; Fig. 5C; Supplementary Data Figs S6 and S7), and that *B. napus* diverged from *Arabidopsis thaliana* at ~14.3 Mya (*K*_s_ peak value ~0.40; Fig. 5C) [1, 11]. Additionally, the identification of up to six homologous copies within a single synteny block confirmed the almost complete triplication and allotetraploidy of the X5A genome in comparison with that of *A. thaliana* (Fig. 5B; Supplementary Data Figs S6 and S7). As a consequence of such duplication, collinearity was observed not only between subgenome homologs, but also within subgenomes (Fig. 1A; Supplementary Data Figs S6 and S7).

### Structural variations associated with flowering time-related genes

Similar to ZS11, X5A is a semi-winter type with a biennial habit but moderate vernalization requirement. It represents the predominant rapeseed type in the Yangtze River Basin of China. However, X5A exhibits earlier flowering and maturity than ZS11 (Fig. 6A). This early maturity trait is inherited by cultivars such as FY737, which were developed using X5A as the maternal parent (Fig. 6A). To analyze how SVs affect early flowering and maturity in X5A, we selected nine other high-quality rapeseed genomes: Darmor, Tapidor, Quinta (winter), ZS11, Gangan, Zheyou7, Shengli (semi-winter), Westar, and No2127 (spring), and compared their SVs with X5A as reference. SV numbers observed among individual accessions varied between 19 152 and 32 755; the spring No2127 and winter Darmor accessions exhibited the highest level of SV compared with the X5A genome (Fig. 6B). Among them, 349–631 SVs were identified in the vicinity of flowering-related genes, with 917 non-redundant flowering-related genes affected by SVs (Fig. 6B; Supplementary Data Table S20). Consistent with the semi-winter characteristics of X5A, the number of SVs between X5A and other semi-winter rapeseed varieties is lower than that between X5A and winter or spring rapeseed varieties (Fig. 6B).

In total, 73 SVs influencing 50 flowering-related genes in spring rapeseed, including *BnaA02.FLC*, *BnaA10.FLC*, *BnaA03.FLOWERING LOCUS R INTERVALOMETER*, *BnaA03.VIN3*, *BnaA04.SUPPRESSOR OF OVEREXPRESSION OF CO 1*, and *BnaA01.CONSTANS-LIKE 9*, may have differentiated spring and semi-winter/winter ecotypes (Supplementary Data Table S20). SVs within *BnaA02.FLC* and *BnaA10.FLC* have been shown to contribute to the diversification of rapeseed ecological types [29]. *BnaA03.VIN3* was proposed as a candidate for ecological type diversification. However, no causal mutations were identified by SNP analysis [30]. Our SV analysis identified a transposon insertion in the promoter region of the *BnaA03.VIN3* in all semi-winter/winter rapeseed, including X5A. This transposon may affect the expression of *BnaA03.VIN3* and could contribute to rapeseed ecotype diversification (Fig. 6C). Meanwhile, 14 genes were affected in all analyzed semi-winter and winter rapeseed, including *BnaC02.FLOWERING LOCUS T*, *BnaC05.SQUAMOSA PROMOTER BINDING PROTEIN-LIKE 10*, *BnaA06.PSEUDO-RESPONSE REGULATOR 5*, *BnaC04.HIGH EXPRESSION OF OSMOTICALLY RESPONSIVE GENES 1* (*HOS1*), *BnaA04.BRAHMA*, and *BnaC02.REPRESSOR OF GA-LIKE1* (Supplementary Data Table S20). Our SV analysis revealed an ~49-kb tandem duplication downstream of *BnaC04.HOS1a*, including two identical copies of *BnaC04.HOS1* (*BnaC04.HOS1b* and *BnaC04.HOS1c*). This duplication event is present in all analyzed semi-winter/winter rapeseeds except X5A, indicating that the low dosage from a single *BnaC04.HOS1* gene is partially responsible for the early flowering of X5A (Fig. 6D) [29, 35, 36]. These genes are potentially introduced from spring-type rapeseed into winter/semi-winter rapeseed, thereby promoting early flowering in X5A, and have potential applications for the breeding of early-maturing semi-winter rapeseed varieties.

## Discussion

We present a gap-free genome for rapeseed elite line X5A with an assembled total genome size of 1004.95 Mb (the estimated size was 1033.31 Mb). Our X5A assembly provides marked improvements in contiguity, completeness, and accuracy over all prior reference genomes. Specifically, all 19 chromosomes were assembled as contiguous sequences, with T2T assembly of eight chromosomes. Increased assembly accuracy can be attributed to the rapid evolution of DNA extraction protocols, advances in sequence data from multiple platforms, more effective technologies, and improved assembly algorithms [15, 24, 34]. The emergence of long-read sequencing technologies has allowed high-quality rapeseed genome assembly. Before our study, based on PacBio data eight rapeseed genomes were assembled *de novo* and had significantly increased contiguity and completeness than assemblies based on short reads [11]. Combining ONT and Bionano optical maps, the Darmor-*bzh* v10 assembly was greatly improved, with a contig N50 of >10 Mb. However, >10% of the sequences remained unassembled, with ~6% unplaced in the latest Darmor-*bzh* v10 assembly [10]. Notably, owing to the low accuracy of the ONT long reads and PacBio CLR, none of the chromosomes had been completely assembled [9–11, 37]. Recent PacBio HiFi reads have achieved >99% accuracy, as they were generated via multi-pass sequencing [38], thereby providing high completeness and contiguity of assemblies of human, rice, and *Arabidopsis* genomes [24, 39]. Here, 9 of 19 rapeseed chromosomes were completely assembled through HiFi reads without human intervention, indicating that HiFi enables efficient assembly of complex genomes, including that of allopolyploids.

The continuous improvement in assemblers has facilitated the generation of complete and contiguous genome assemblies [40]. In particular, hifiasm has recently been used to assemble several plant genomes, including those of rice, strawberry, and tomato, in a gapless manner [34, 41, 42]. Herein we found that integrated ONT and HiFi reads using hifiasm outperformed assembly with HiFi alone. Hifiasm integrated both the ONT and HiFi reads, leveraging their respective strengths in long-range information and high accuracy. Our workflow produced fewer short fragmented contigs, with a contig number of 651, which was less than that of HiFi alone (891). Moreover, integrated ONT and HiFi reads using hifiasm distinctly enhanced the contig N50; that of the mixed assembly was 50.7 Mb higher than that of the HiFi assembly alone (31 Mb). Therefore, continuous optimization of assembly algorithms is important for genome assembly.

Our gap-free, near-complete, allopolyploid oilseed rape genome provides novel insights into the genomes of Brassicaceae, especially their highly repetitive and similar regions, including centromeres [20, 24, 27]. With the development of sequencing technologies, long-read sequencing, especially PacBio HiFi and ultra-long ONT sequencing, has enabled complete assembly of centromeric regions. Based on the T2T genome in humans, the genomic and epigenetic landscapes of centromeres were completed. Our analysis revealed large and unexpected genetic variations in centromeric regions, including multi-megabase structural variants, which may be strongly associated with important agronomic traits [27]. Using two T2T assembly genomes for rice, Song *et al*. found that *CentO* satellite motifs are highly conserved among centromeres [24]. Studies on centromeres in human and model plants have demonstrated that these regions can be studied using modern sequencing technologies. The rapeseed genome was first released in 2014, and the centromeres were identified; however, they were not well assembled owing to their high complexity. We observed high collinearity between X5A and ZS11_HZAU genomes, except in their centromeric regions [19]. ONT raw reads were evenly covered with no apparent breakpoints in all centromere regions, indicating that the centromeres in the X5A assembly are highly contiguous and complete. Meanwhile, the X5A assembly had longer centromeres than the ZS11_HZAU assembly in most chromosomes, indicating a better assembly of repeat sequences in the centromere region of X5A [11, 34]. The well-assembled centromeres revealed in this study provide valuable resources for comprehensive analysis of centromere function.

The Yangtze River Basin serves as the primary region for rapeseed cultivation in China, where semi-winter rapeseed is the predominant variety. Paddy upland rotation is the predominant farming practice employed for rapeseed production in this area. However, in recent years, conflicts have arisen in rapeseed production owing to the cultivation of double-season rice and the extended growth period of intermediate-maturing rice. To address this, the introduction of early-maturing rapeseed cultivars has been proposed to alleviate the conflicts between double-season rice and rapeseed. This approach would facilitate the establishment of a ‘rice–rice–rapeseed’ pattern to more effectively utilize winter-fallow land [43]. The potential of rapeseed as a horticulture crop has also been explored, and early-flowering rapeseed varieties possess enhanced horticultural value [5]. Many loci that contribute to flowering time and ecological types have been identified; however, their functional characterization has been limited to only a few cases.

Gap-free genomes offer an unprecedented opportunity for more comprehensive identification of previously hidden SVs in complex genomes [20, 44]. Taking advantage of our gap-free X5A reference genome, 917 non-redundant flowering-related genes affected by SV were identified, including a transposon insertion in the promoter of *BnaVIN3.A03*. Previous studies have indicated that *BnaVIN3.A03* was significantly associated with flowering time and ecotype diversification; however, to date, causal mutations have not been identified using SNP analysis [30]. The identified insertion is present in all semi-winter rapeseed varieties analyzed in this study, including X5A, suggesting its potential contribution to the differentiation between spring and semi-winter/winter ecotypes. We can infer that *BnaVIN3.A03* and *FLC*s determine the semi-winter ecological type of X5A, enabling its adaptation to the environmental conditions of the Yangtze River Basin [11, 29]. Moreover, the identified 14 genes exhibited SVs between X5A and other analyzed semi-winter and winter rapeseed, including copy number variation in *BnaC04.HOS1*. These genes and SVs may play crucial roles in regulating early flowering and early maturity traits in X5A. In comparison with *BnaVIN3.A03* and *FLC*s, which directly determine the differentiation of ecological types, these genes with relatively weaker effects have higher potential for breeding of early-maturing rapeseed in the Yangtze River Basin.

## Conclusion

In conclusion, we assembled and revealed the first gap-free rapeseed genome by integrating PacBio HiFi, ONT ultra-long reads, and Hi-C technology. This assembly not only enables further understanding of the allopolyploid oilseed rape genome but also improves our ability to identify agronomic trait-related genes and variants, including those involving complex copy number/structural variations.

## Materials and methods

### Plant materials, DNA extraction, and sequencing

X5A, which is a shared paternal pure line of several elite hybrid varieties, including FY737, was chosen for genome assembly. The X5A plants were grown in a greenhouse under a 16-h light and 8-h dark cycle at 22°C for 10–12 days. Total genomic DNA was obtained from leaf tissue of X5A using the CTAB method, and Hi-C sequencing libraries were prepared using the NEBNext Ultra II DNA library preparation kit and DpnII enzyme (Ipswich, MA, USA). The SMRTbell libraries were prepared using 15-kb preparation solutions from Pacific Biosciences for HiFi sequencing, and HiFi reads were produced using the CCS software (https://github.com/pacificbiosciences/unanimity) with the parameters --min-passes 3 --min-snr 2.5 --top-passes 60. Genomes were sequenced on the Nanopore PromethION 48 and Illumina Novaseq 6000 platforms using standard ONT and Illumina protocols. Paired-end libraries were prepared using standard Illumina protocols with an insert size of 350 bp, and 150-bp reads were generated for each individual. Illumina reads were used to estimate genome size and heterozygosity using Jellyfish (v2.2.10, parameter: jellyfish histo-h Max_count) and GCE (v1.0.0, https://github.com/fanagislab/GCE).

### Sequencing data filtering

ONT ultra-long raw sequencing reads were filtered to remove unreliable data (sequences with average QVs <7). Data were filtered for fragments <10 kb using Filtlong (v0.2.4, https://github.com/rrwick/Filtlong), and only the valid data (pass reads) were used for subsequent analysis. Connector sequences were filtered using Porechop (v0.2.4; https://github.com/rrwick/Porechop), and Filtlong was applied to filter the data further, with a length threshold of ≥30 kb and mean read quality scores of >90%, which were used for the assembly.

PacBio HiFi raw sequencing data (subreads) were filtered using the software ccs (v6.0.0; parameters: -min-passes snr 2.5 -top-passes 60; https://github.com/PacificBiosciences/ccs) to evaluate the quality of the raw data. Data with fewer than three laps of sequencing and low-quality subreads with SNR <2.5 were filtered, and valid data were obtained for subsequent analysis.

The Hi-C raw reads underwent filtering using fastp (https://github.com/OpenGene/fastp) to remove low-quality and spliced sequences. The resulting clean data were compared with the reference genome and further filtered using HICUP (v0.8.0; http://www.bioinformatics.babraham.ac.uk/projects/hicup/) based on the following criteria: removal of unmapped reads that did not uniquely match the reference genome at both ends; removal of invalid pairs, including Self Circle and Dangling End; and removal of duplicate sequences resulting from PCR amplification.

### Genome assembly

NextDenovo (v2.5.0) (https://github.com/Nextomics/NextDenovo), Flye (v2.9-b1768) [45], and Canu (v2.2) [46] were used for ONT data assembly [22]. NextDenovo was applied with the parameters: read_cutoff = 1k, seed_cutoff = 44382, blocksize = 1g, nextgraph_options = -a1. Flye release was set with –genome-size 1000 m. Canu was used to assemble the genomes using default parameters. HiFi reads were assembled using hifiasm (−0.16.1-r578, https://github.com/chhylp123/hifiasm) with default parameters to generate a draft contig genome [41]. Hifiasm was used to integrate HiFi and ONT reads (−0.19.5-r578, −t 64 -l 0 —ul) [41]. The clustered contigs were ordered and oriented using ALLHiC, a software tool specifically designed for scaffolding genome assemblies using Hi-C data (https://github.com/tangerzhang/ALLHiC).

Three levels of sequences were used to fill the gaps, with the following priority: NextDenovo, NECAT, hifiasm, Flye-corrected assembly > ONT data > HiFi data. For each gap region, the sequences from the three levels were aligned to the genome, including the gap and its flanking regions extended by 5–20 kb. If the aligned position spanned both ends of the gap, the best alignment region with the longest length was selected to replace the gap region in the genome using the gap-filling data. This alignment was performed using Winnowmap v1.11 with the parameters k = 15 and —MD. Then the genome, original HiFi/ONT data, and other assembly versions were aligned to the filled genome. The alignment of reads at the breakpoint regions was examined to verify the reliability of the filled gaps using minimap2 v2.17-r941.

For error correction, the HiFi reads with a length of ≥10 kb were aligned to the gapped version of the genome using Winnowmap2, with the parameters k = 15, greater-than, distinct = 0.9998, —MD, and -ax map-pb. The aligned fragments were filtered using SAMtools view (v1.10) with the parameter -F 256. Chimeric alignments were removed using falconc bam-filter-clipped with the parameters -t and -F 0x104. With the filtered alignment information, a specialized branch of racon (v1.6.0, −L, −u) was used for error correction (https://github.com/isovic/racon/tree/liftover).

To identify telomeres, all ONT reads were aligned to the reference genome using Winnowmap (v1.11) with the parameters k = 15 and —MD. Reads that aligned within 50 bp of the chromosome ends were collected. The occurrence of telomeric repeats (CCCTAAA/TTTAGGG) within each read was calculated using the Telomerase database (http://telomerase.asu.edu/sequences_telomere.html). TeloExplorer modules in quarTeT (user-friendly web toolkit) were utilized to pinpoint potential telomeres and determine their locations on each chromosome [47]. The read with the highest occurrence was defined as the reference, while the remaining reads were considered as queries. The medaka_consensus tool (v1.2.1) with the parameters -m r941_min_high_g360 (https://github.com/nanoporetech/medaka) was used to query and reassemble the telomeric reads. The best alignment results were used to patch and extend the chromosome ends. Telomeric (HiFi and ONT) reads were manually identified, and the telomere-missing chromosomes were patched with the telomeric sequences. Continuous clusters with candidate centromeric tandem repeats were identified based on the typical characteristics of centromeric regions, which include a high density of short tandem repeats and low density of genes.

### Assembly validation

We assessed the assembly completeness and sequencing uniformity by mapping clean reads to X5A assembly using BWA (v0.7.8, with the parameters mem -t 4 -k 32 -M) [48], and calculating the mapping rates and coverages using SAMtools (with the -a flag to include sites with no coverage) [49]. The completeness of gene regions was assessed by BUSCO (4.0.5) with the embryophyta_odb10 dataset (2019-11-20) [50]. HiCExplorer (v3.6) was used to align the Hi-C data to the X5A assembly [51]. The output matrix file was visualized using HiCExplorer (v3.6) [52]. In addition, the quality of the assembly was assessed using the Merqury quality value, which was based on a 19-mer ‘hybrid’ Merqury *k*-mer database that combined Illumina PCR-free reads [53]. After identifying LTR structures and using complete LTR elements to calculate the LAI value, we performed calculations to determine the genome assembly integrity, which was quantified using the LAI score (https://github.com/oushujun/LTR_retriever).

### Genomic comparisons and synteny analysis

We used MUMmer version 4.0.0 (https://github.com/mummer4/mummer) to perform sequence comparisons between the X5A assembly and previously published high-quality genomes. Then, syri was used to identify conserved regions and detect structural rearrangements between the genomes (v1.6; https://github.com/schneebergerlab/syri). After MUMmer alignment, regions of the genome that did not align to ZS11-HZAU were considered as newly assembled regions, and the genes contained within these regions were considered as novel genes [54]. To characterize the functions of newly identified genes in X5A, we conducted GO analysis using InterProScan 5 (v5.47) to classify genes into biological processes, cellular components, and molecular function terms. The R package clusterProfiler was utilized for GO enrichment analysis and visualization.

### RNA extraction, library construction, and transcriptome sequencing

To annotate the genome, six tissue types (leaf, flower, stem, root, silique, and stem tip) were collected at two developmental stages: seeding and flowering. Total RNA was extracted utilizing a kit (Tiangen, China) and evaluated for quality using agarose gel electrophoresis, NanoDrop spectrophotometry, and Qubit fluorometry. Full-length cDNA libraries were prepared using the ONT kit (SQK-PCS109) and sequenced on the PromethION 48 platform (PAE33370). Base calling was performed using Guppy 5.0 software (Oxford Nanopore), and the sequences were filtered using the NanoFilt tool (length >50, quality >7) to obtain clean data (https://github.com/wdecoster/nanofilt).

### Gene annotation

A hybrid gene annotation pipeline was employed to annotate protein-coding genes in X5A, which integrated homology-based, *de novo*, and transcriptome-based prediction methods. Four plant genomes, namely *A. thaliana* (https://ftp.ncbi.nlm.nih.gov/genomes), *B. napus* (http://cbi.hzau.edu.cn/bnapus/), *B. oleracea*, and *B. rapa* (http://brassicadb.cn/#/Download/) were used as the reference genomes. Protein sequences from these genomes were aligned to the X5A genome using the TBLASTN program. Exonerate (v2.4.0, parameters: -model protein2genome –showtargetgff 1) [55] was used to predict the transcripts and coding sequences based on the alignment results.

Transcriptome prediction was based on ONT sequences. Data were filtered using the NanoFilt software (v2.8.0) with the following parameters: -q7 -I100 –headcrop 30 –minGC 0.3. Subsequently, full-length sequence identification was performed using the Pychopper software (v2.7.2) with the following parameters: -r report.pdf -u unclassified.fq -w rescued.fq. The obtained full-length sequences were corrected using the racon software (v1.4.21) based on the original reads, with the parameter -t16 -q7. The corrected full-length sequences were aligned to the genome using the minimap2 software (v2.17-r941) with the parameters -ax map-ont -xsplice -G 1000000. The alignment results in BAM format were reconstructed into transcripts using the StringTie software (v2.1.4) with the parameters -p 15 -R -L. The predicted coding regions were then identified within the predicted transcripts using TransDecoder software (v5.1.0) with the parameter -S.

*Ab initio* gene predictions in the repeat-masked genome were performed using Augustus (v3.3.2) [56], GENSCAN (v1.0) [57], and GlimmerHMM (v3.0.4) [58]. The MAKER (v2.31.10, parameters: maker_exe.ctl maker_opts.ctl maker_bopts.ctl -ignore_nfs_tmp -fix_nucleotides; except for input/output paths, default settings were used for other parameters) software was used to integrate gene sets predicted by various methods [59]. EST evidence was used as input for transcriptome annotation, protein homology evidence was used as input for homology-based prediction, and the merged *de novo* annotation results were used as input for gene prediction. The final set of genes was obtained by validating the predictions from various aspects, such as the accuracy of the ORF region, correctness of the start and stop codons, and gene-length filtering. Multiple evidence-supported genes were identified by comparison and used to replace the existing MAKER annotation (v2.31.10, parameters: maker_exe.ctl maker_opts.ctl maker_bopts.ctl –ignore_nfs_tmp -fix_nucleotides; In the three configuration files for MAKER, only the input and output paths were modified; all other parameters were set to default). Additional single-evidence results displaying minimal overlap with the existing gene sets were added based on the estimated number of genes in the following order: transcriptome evidence, homology evidence, and *de novo* evidence.

### Repeat annotations

The *de novo* method was used to predict model sequences based on the genome sequence itself utilizing RepeatModeler software (version: open-1.0.11; parameters: BuildDatabase -name mydb; RepeatModeler -database mydb -pa 10) (https://github.com/Dfam-consortium/RepeatModeler). LTR sequences were predicted using the LTR_FINDER software (version: Official release of LTR_FINDER_parallel; parameters: -threads 16 -harvest_out -size 1 000 000 -time 300), and LTR sequences were obtained using the LTR_retriever software (version: 2.9.0; parameters: -threads 16) to remove redundancy from the results predicted by LTR_FINDER. The two *de novo* sequence sets were merged to obtain the *de novo* repeat sequence library, which was then merged with the RepBase library (version: 20181026) and analyzed using the RepeatMasker software (version: open-4.0.9, parameters: -nolow -no_is -norna -parallel 2) (https://github.com/rmhubley/RepeatMasker) to predict repeat sequences, resulting in De novo + RepBase results. The RepeatProteinMask subprogram of the RepeatMasker software (version: 4.0.9; parameters: -noLowSimple -pvalue 0.0001) was used to predict repeat sequences of the TE protein type, resulting in TE protein results. Finally, all repeat prediction results were merged, redundancy was removed to obtain the final genome repeat sequence set, and TE results were combined.

### Evolutionary analysis

Gene family clustering was performed using OrthoFinder (version: 2.3.12; parameters: -M msa) [60]. The software Muscle (v3.8.31) [61] was used to perform multiple sequence alignments on the protein sequences of each single-copy gene family. We used trimAl (v1.2rev59; parameters: -gt 0.2) [62] to filter the comparison results, and the filtered comparison results were merged and connected as a supergene. The maximum likelihood phylogenetic tree was generated using RAxML v8.2.10 with the PROTGAMMAILGF model, and bootstrap analysis with 1000 replicates was performed. Based on the topology of the phylogenetic tree and table of fossil time nodes (as above), the mcmctree subroutine of the PAML package (v4.9; parameters: nsample = 3 000 000; burnin = 8 000 000; seqtype = 0; model = 4) was used to estimate the differentiation times of the selected species time [63]. The CAFE (v3.1) [64] software was used to estimate the number of gene family members for each branch’s ancestor using a birth–mortality model based on species evolutionary trees and gene family clustering results to predict the contraction and expansion of the species’ gene family relative to the ancestor. Proteins from different species were aligned using BLAST (v2.6.0+; parameters: -evalue 1e-5 -outfmt 6), followed by MCScanX [65]. We used the yn00 module in PAML [63] to calculate the frequency of synonymous and non-synonymous (*K*_a_) mutations, and the ratio of non-synonymous to synonymous mutation rate (*K*_a_/*K*_s_) for co-linear gene pairs. Additionally, we used the gff3 annotation file and JCVI to determine whether similar gene pairs were in proximity on the chromosome, enabling us to identify all the genes in the covariance block [66].

For karyotype analysis, to identify collinearity between the protein sequences of five selected species and those of the AEK ancestor, we used the SynOrths software (v1.0; available at http://brassicadb.cn:82/download_genome/tools/SynOrths/SynOrths_V1.0.tar.gz) with the following parameters: -m 20 -n 100 -r 0.2. Based on the collinearity blocks identified between each species and the AEK ancestor, we analyzed the positional distribution of each collinearity block within each species. Color-coded bars were used to represent the collinearity blocks from the five species in a bar plot PDF file, with different colors corresponding to different chromosomes in the AEK ancestor. The bar plot was integrated and drawn, and the phylogenetic tree illustrating species divergence and divergence time was drawn using Adobe Illustrator to visualize the results.

### Structural variation identification

To identify SVs between X5A and nine other high-quality-assembled rapeseed genomes, we aligned the nine genomes to X5A using the minimap2 alignment tool with the parameters -ax asm5. The resulting alignments were analyzed using syri (—nc 5 —invgaplen 20 000) to call SVs. To analyze how SVs affect earlier flowering time in X5A, we screened for SVs within the flowering genes and within a 5-kb region upstream and downstream of the genes [11, 67]. Subsequently, the frequency of each gene in different ecotypes (spring, winter, and semi-winter) was computed using the IF function in Microsoft Excel (Microsoft Corp., Redmond, WA, USA).

## Acknowledgements

This work was supported by the Hunan Province Science and Technology Innovation Plan Project (grant number 2021NK1004), the Changsha Natural Science Foundation (grant number kq2208157), and China Agriculture Research System of MOF and MAR (grant number CARS_12). The authors thank Wuhan Benagen Tech Solutions Co., Ltd for assistance with bioinformatics analysis.

## Author contributions

B.L. performed the research and wrote the paper. L.Q., L.F., L.D., and R.H. participated in discussions and provided valuable advice. L.Y., Y.G., D.G., X.L., X.Z., and Q.Y. analyzed the data. M.L., T.W., and M.Y. designed and led this project.

## Data availability

The genome and RNA-seq data for X5A have been deposited in the National Center for Biotechnology Information (https://www.ncbi.nlm.nih.gov/) under the BioProject accession number PRJNA950196. Additional material generated during this study can be obtained from the corresponding author upon reasonable request.

## Conflict of interest

The authors declare no conflict of interest.

## Supplementary data

Supplementary data is available at *Horticulture Research* online.

## Supplementary Material

Web_Material_uhad171
