# Supplementary material for: A gap-free reference genome reveals structural variations associated with flowering time in rapeseed (Brassica napus)
Source: Hortic Res. 2023 Aug 29;10(10):uhad171. doi: 10.1093/hr/uhad171 (PMC10569240; doi:10.1093/hr/uhad171)

Gap1: Checking the gap regions by HiFi

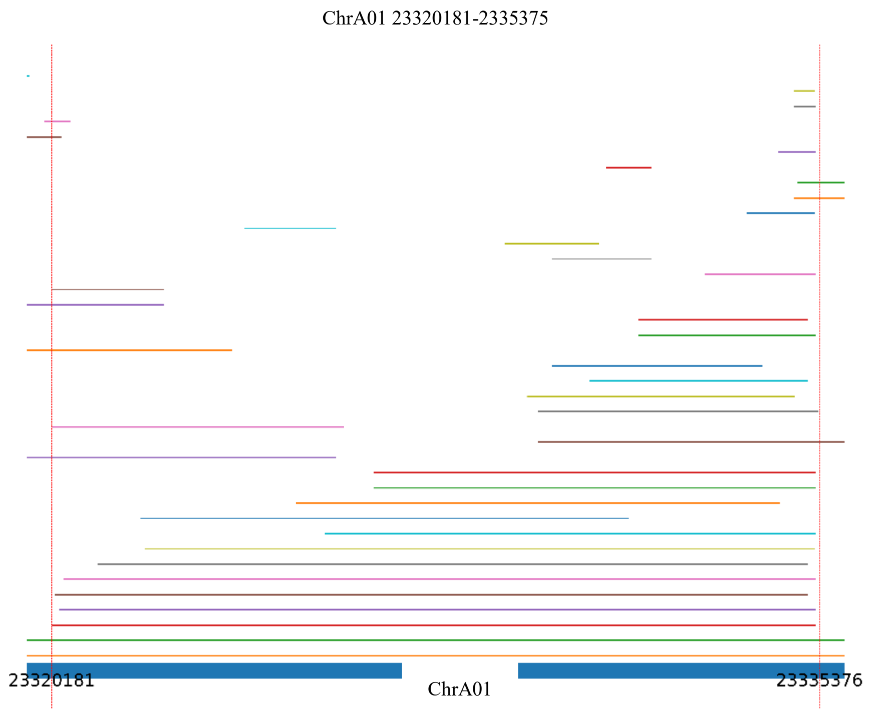

Gap2: Checking the gap regions by Flye

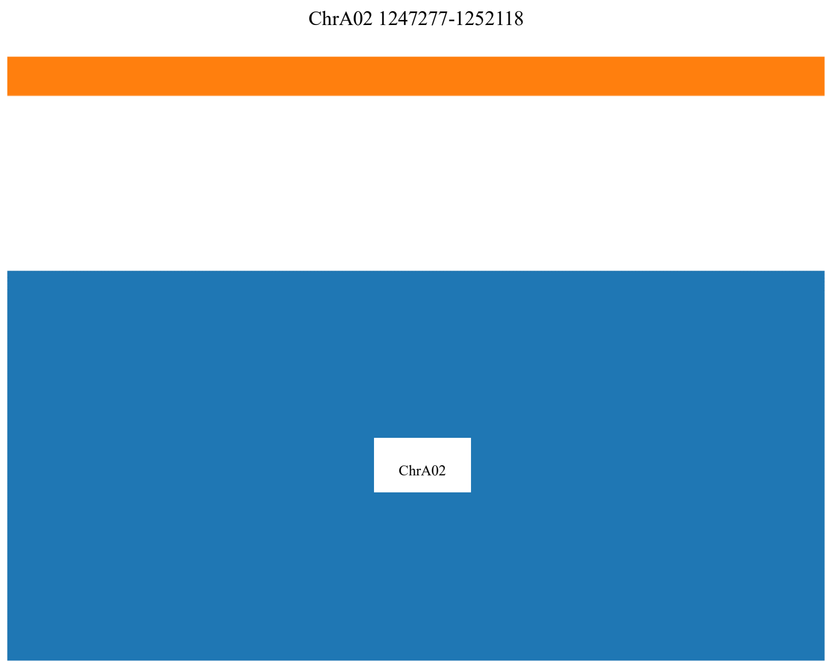

Gap2: Checking the gap regions by Hifiasm

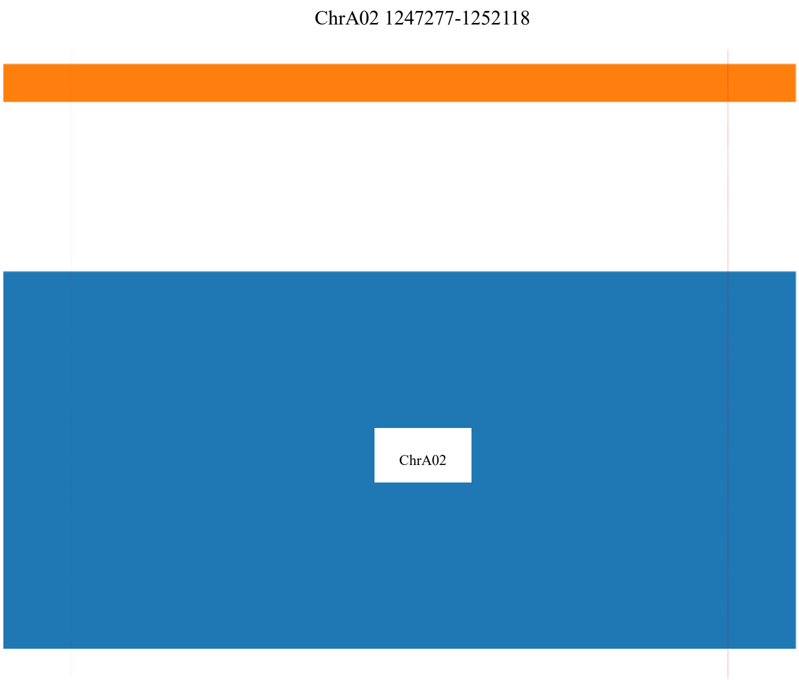

Gap2: Checking the gap regions by Necat

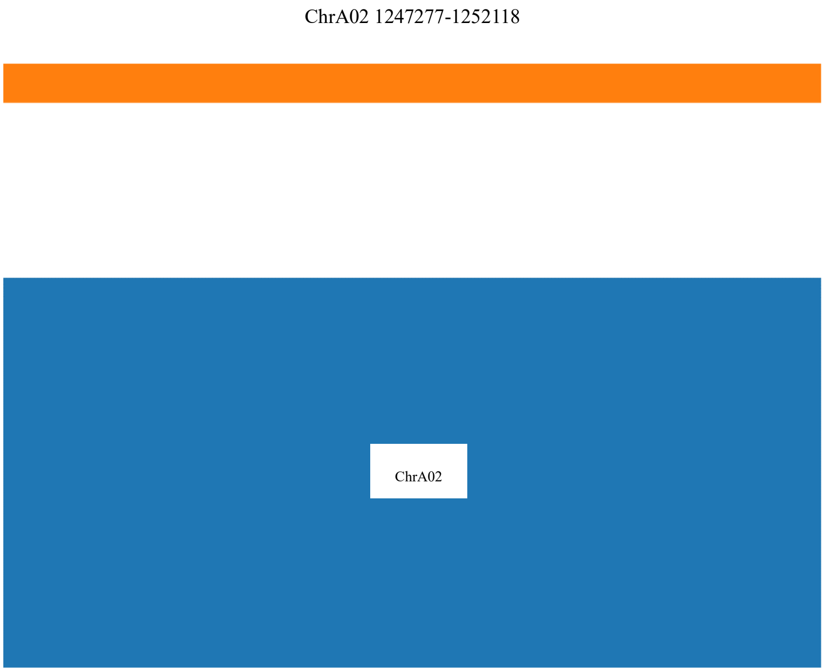

GAP3: Checking the gap regions by HiFi

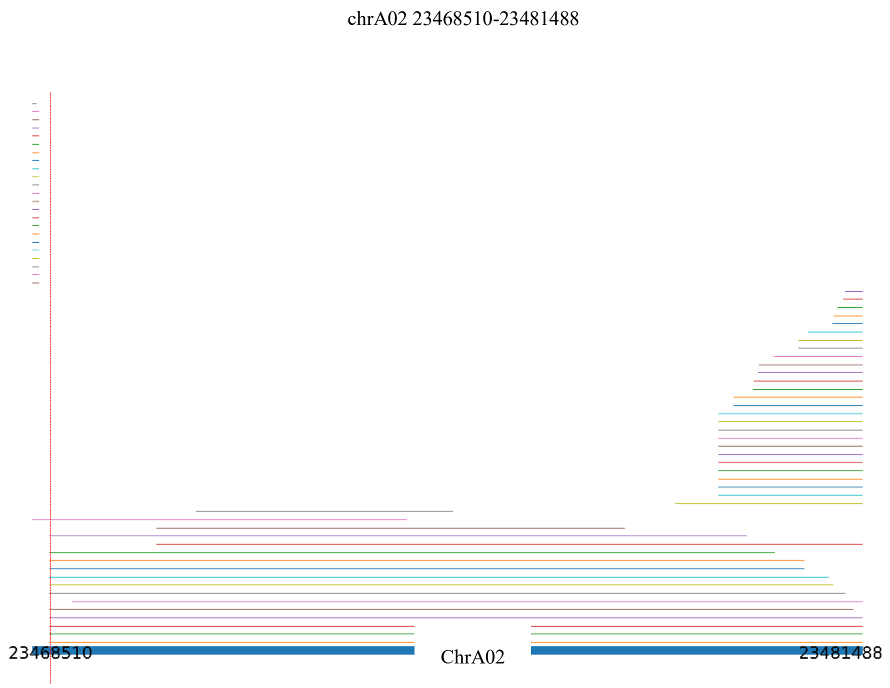

GAP3: Checking the gap regions by Hifiasm

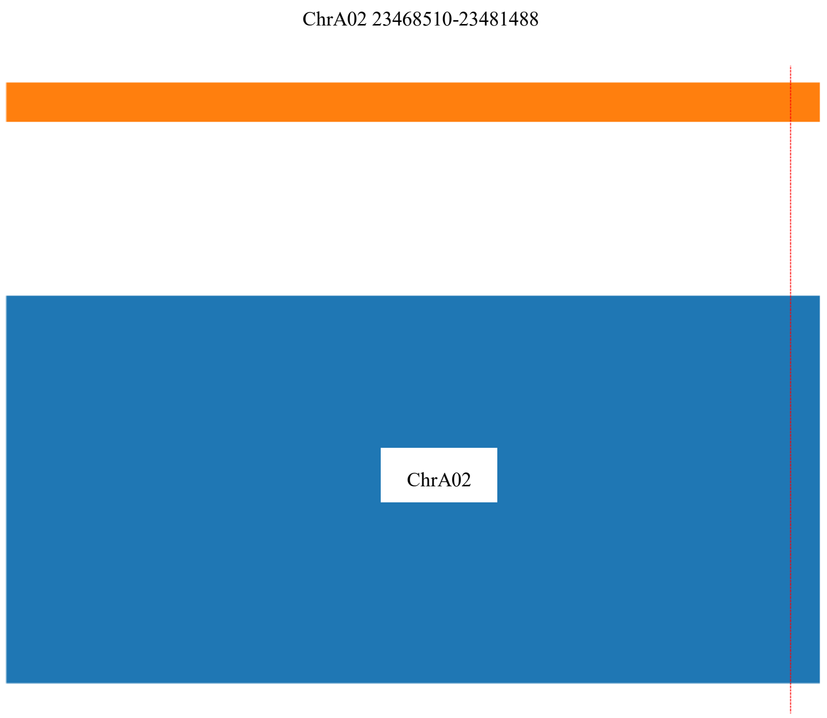

Gap3: Checking the gap regions by Necat

ChrA02 23468510-23481488

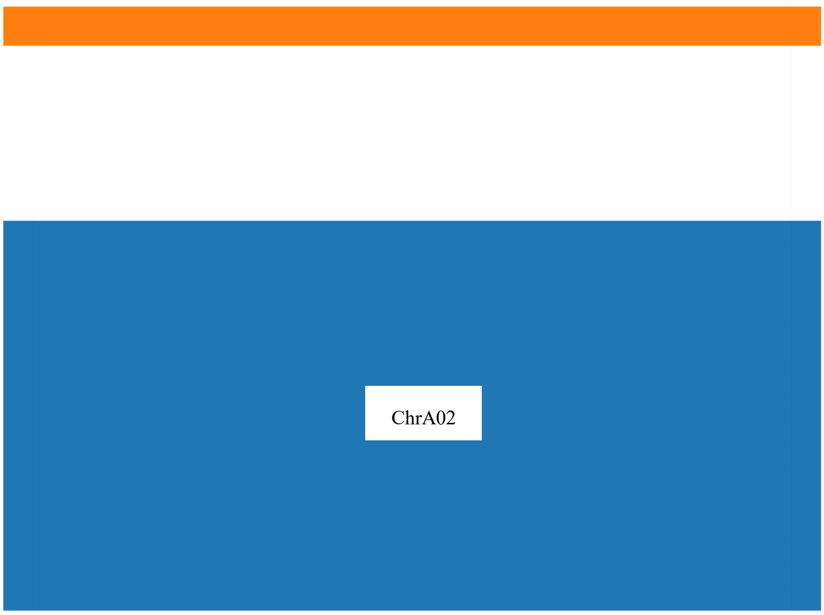

Gap4: Checking the gap regions by Flye

ChrA04 24913412-24999489

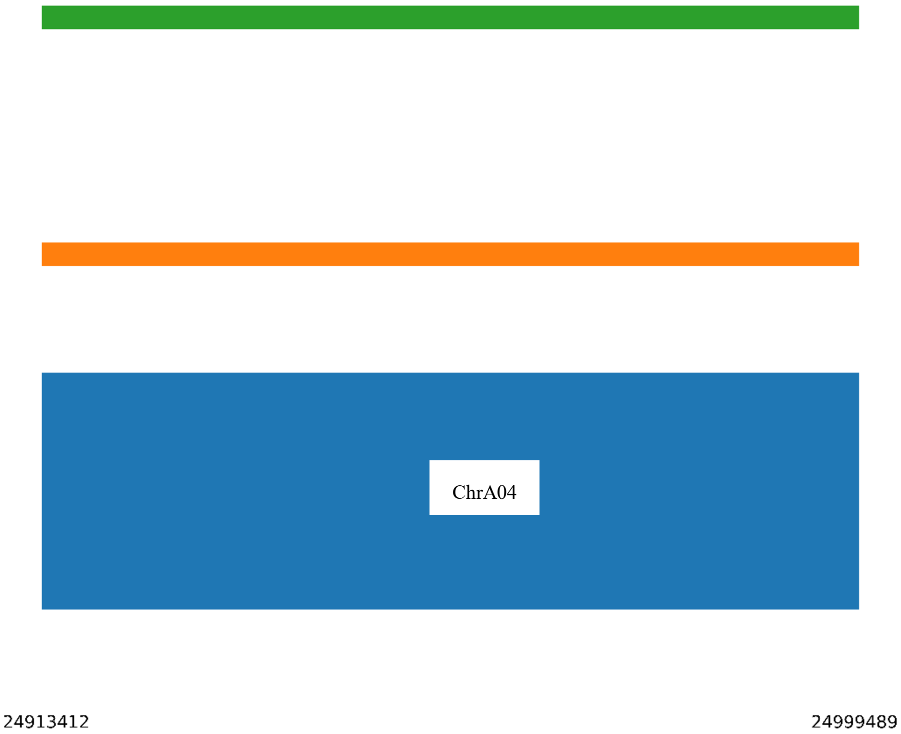

Gap4: Checking the gap regions by Nextdenovo

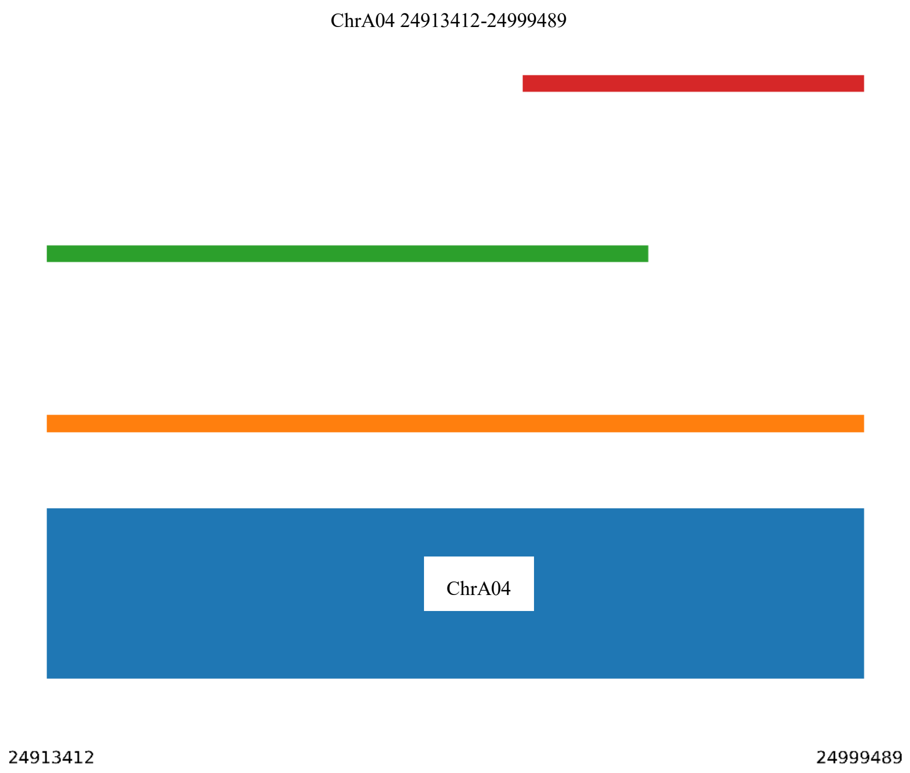

Gap5: Checking the gap regions by Nextdenovo

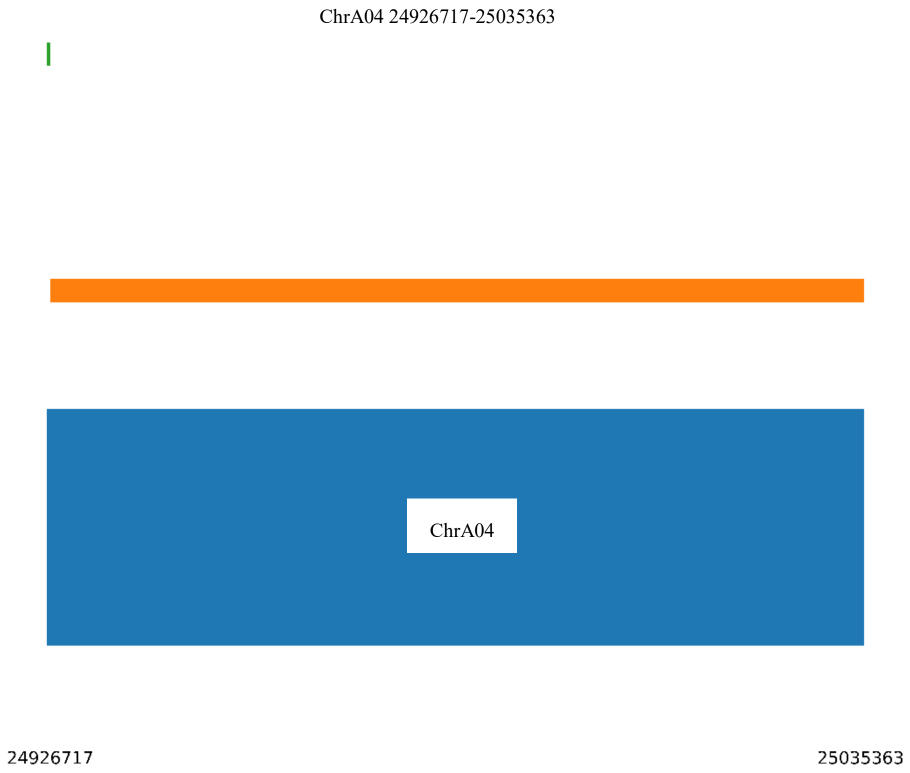

Gap5: Checking the gap regions by Flye

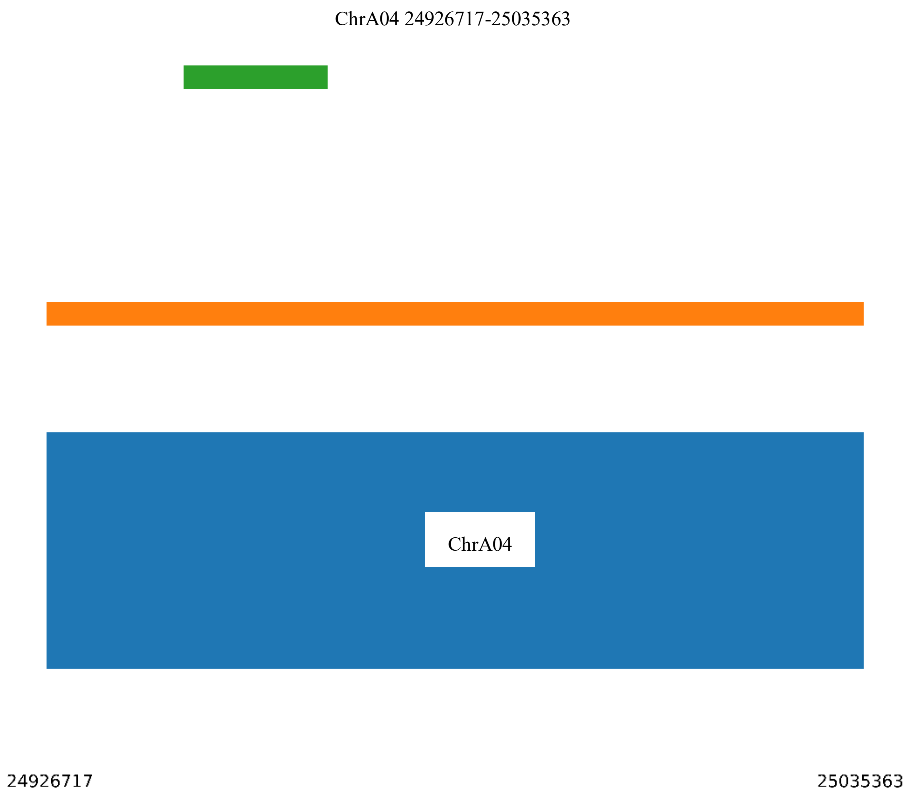

Gap6: Checking the gap regions by Flye

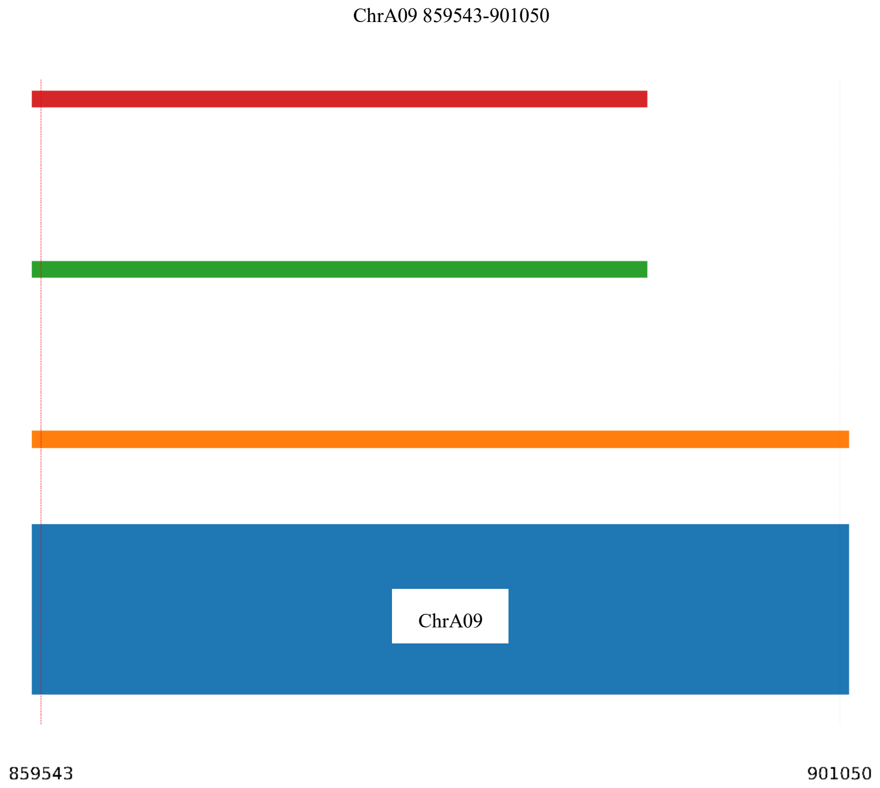

Gap6: Checking the gap regions by Nextdenovo

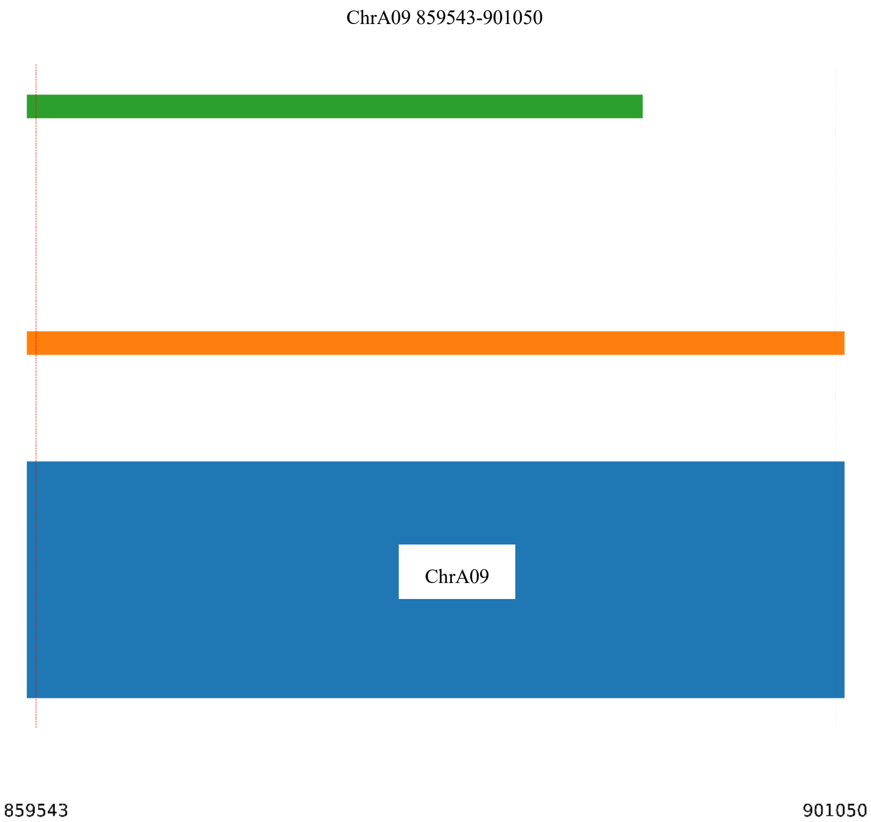

Gap7: Checking the gap regions by ONT

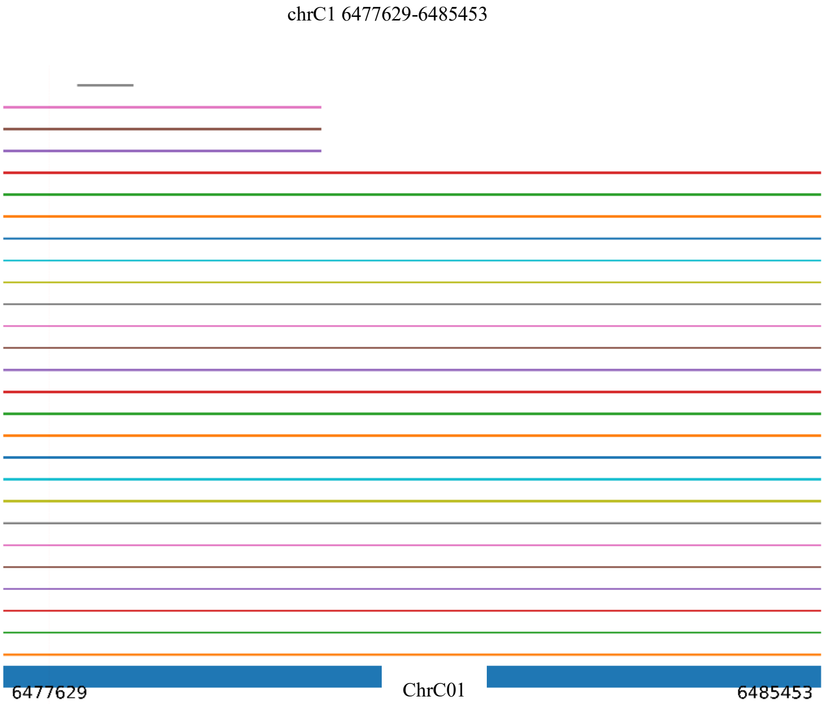

Gap7: Checking the gap regions by Flye

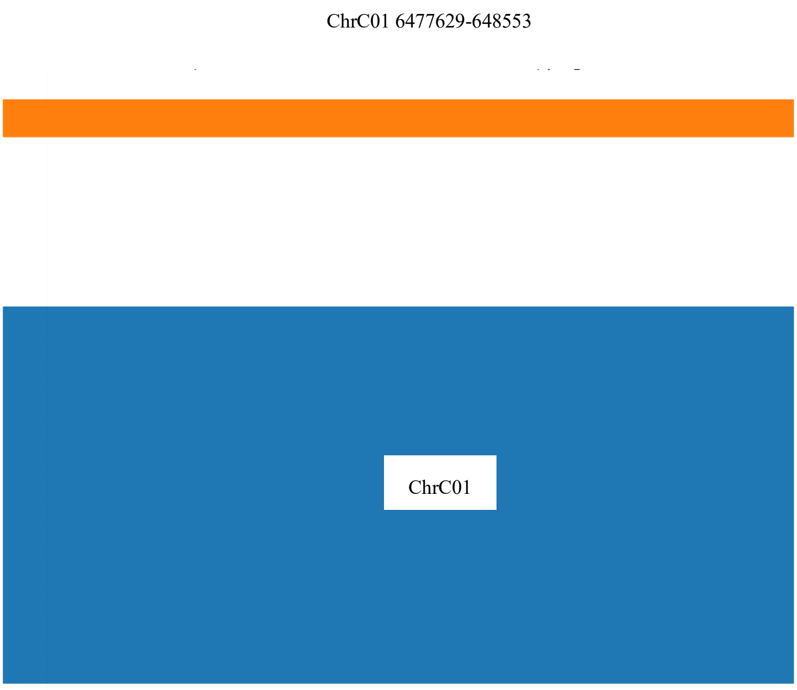

Gap7: Checking the gap regions by Necat

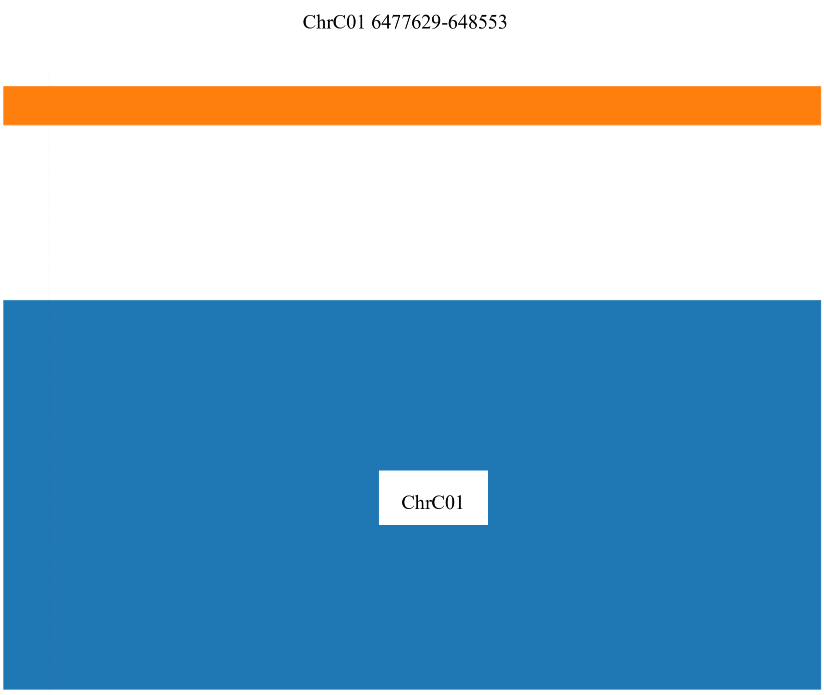

Gap8: Checking the gap regions by Flye

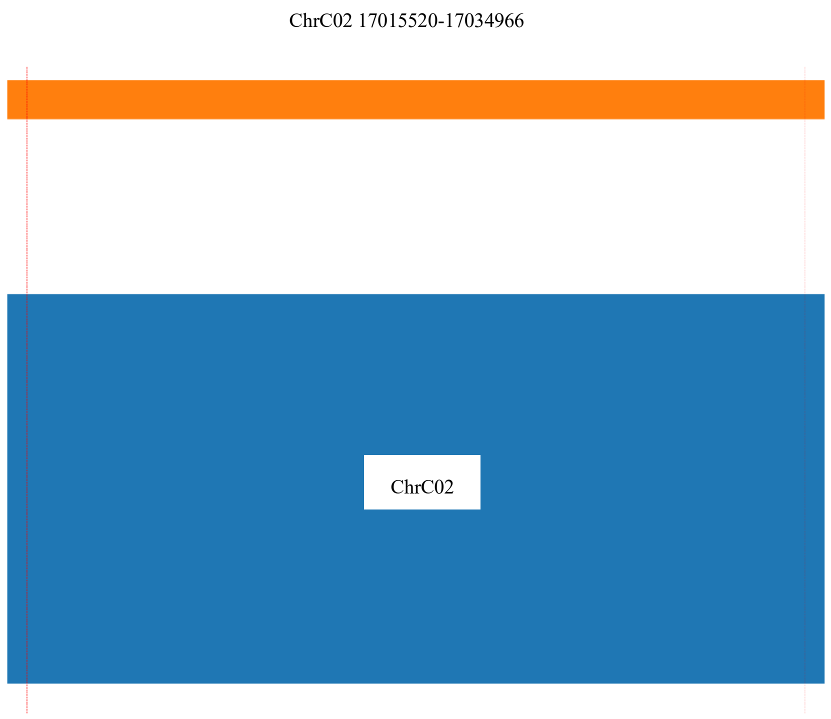

Gap8: Checking the gap regions by HiFi

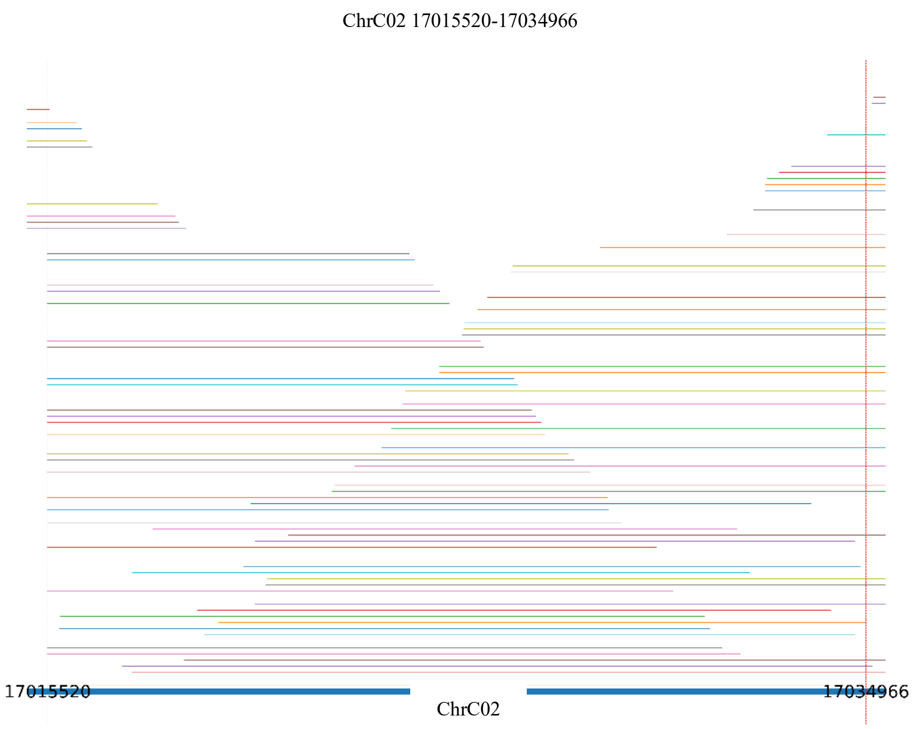

Gap8: Checking the gap regions by Hifiasm

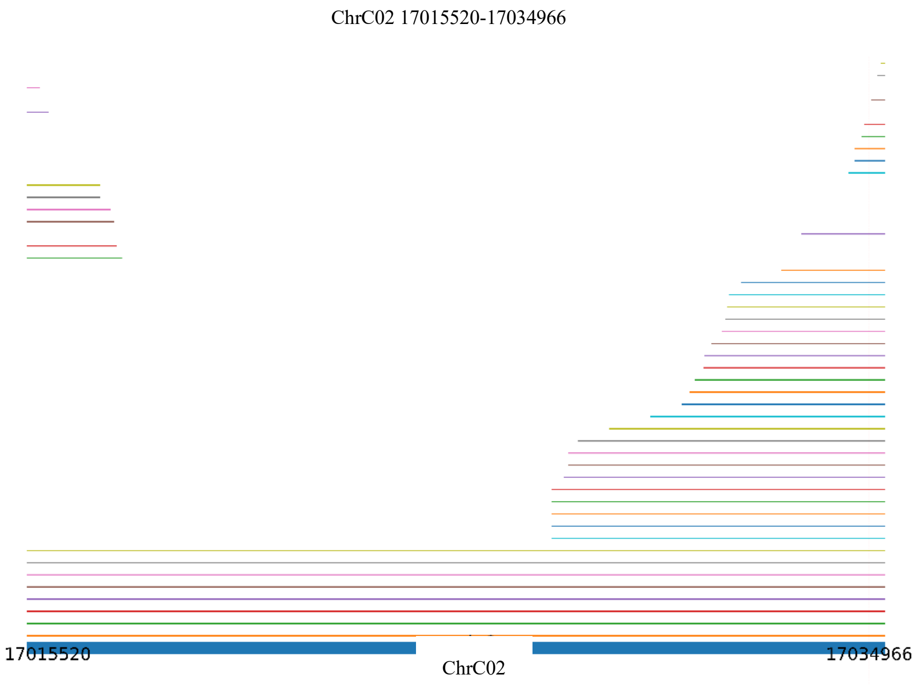

Gap9: Checking the gap regions by Hifiasm

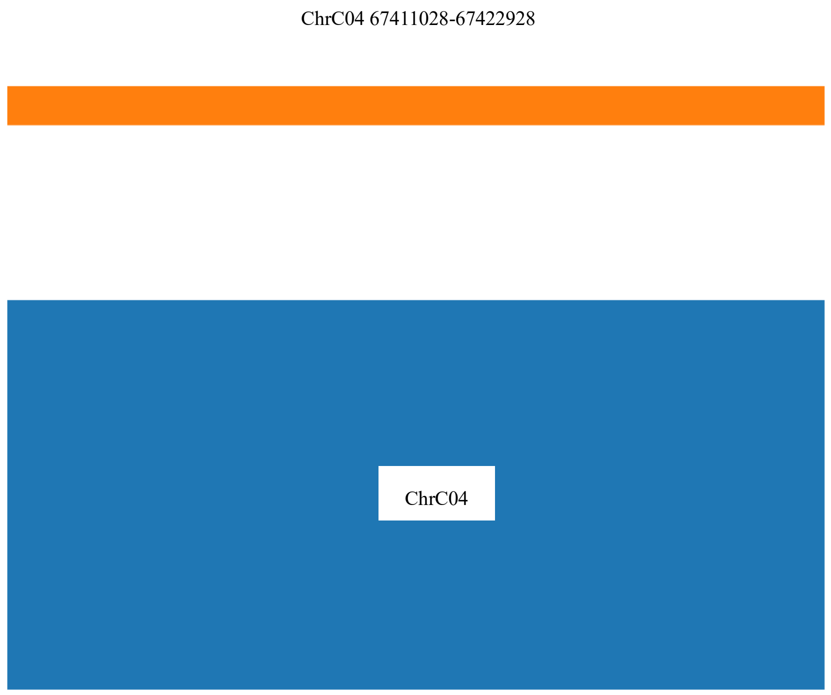

Gap10: Checking the gap regions by Hifiasm

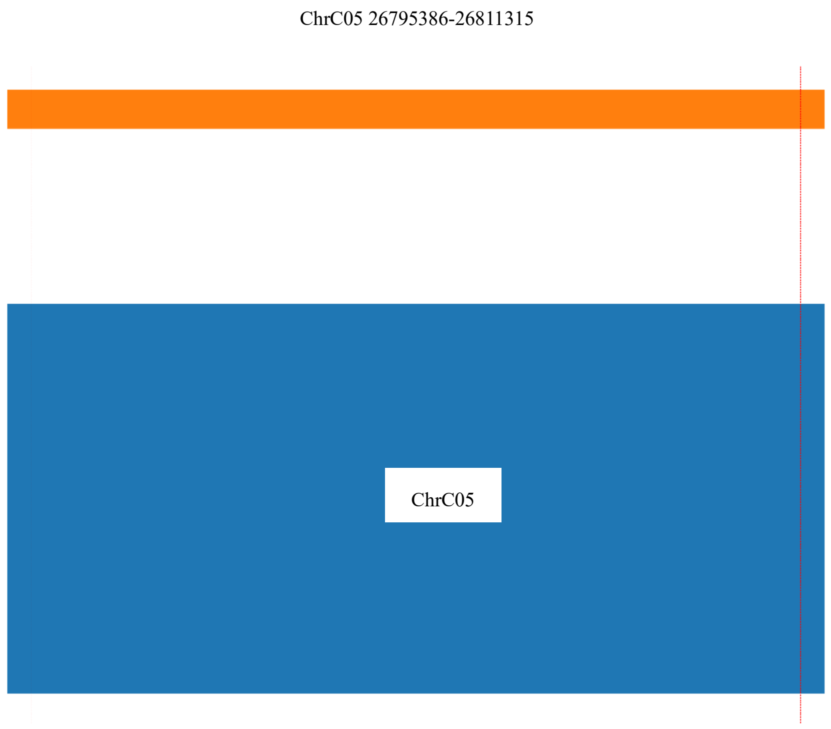

Gap10: Checking the gap regions by Necat

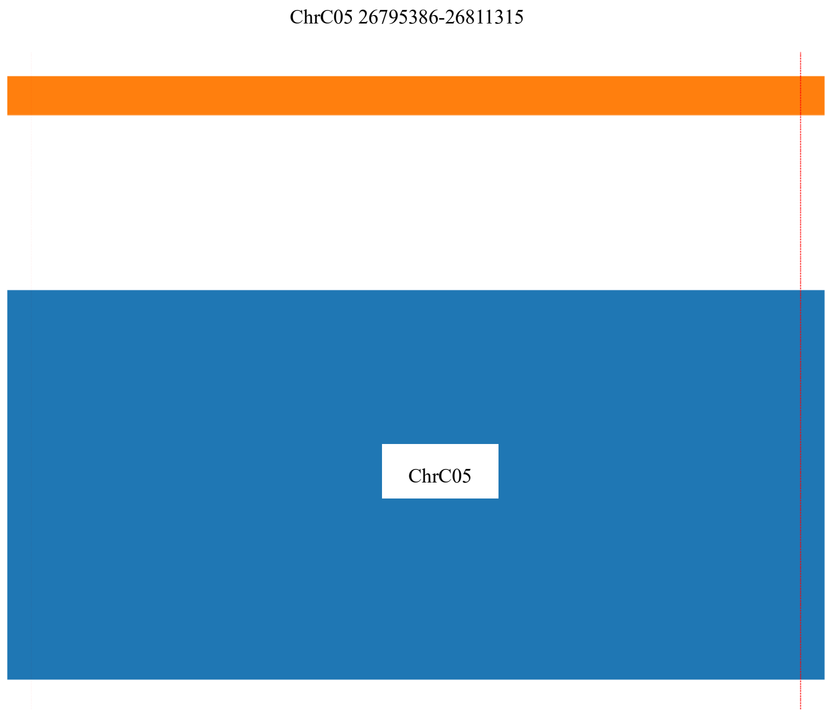

Gap11: Checking the gap regions by Flye

ChrC08 52506196 52513725

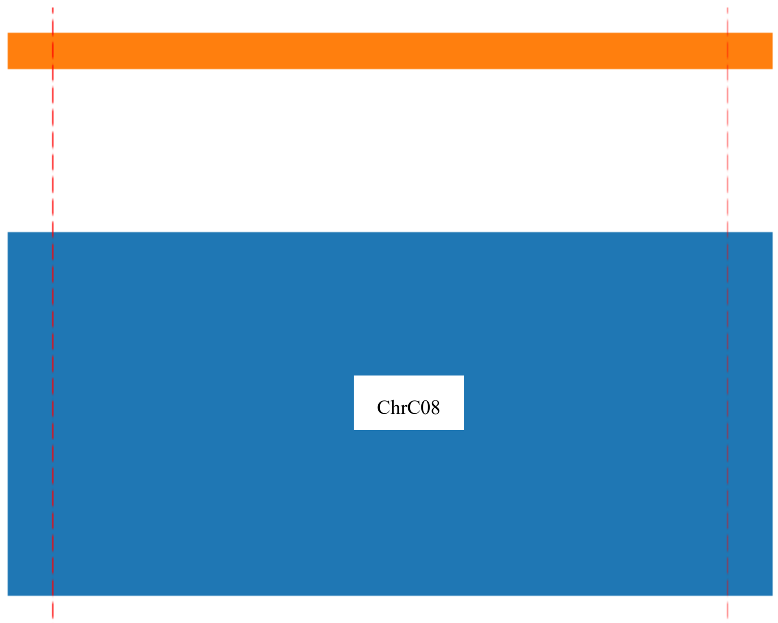

Gap11: Checking the gap regions by Hifiasm

ChrC08 52506196 52513725

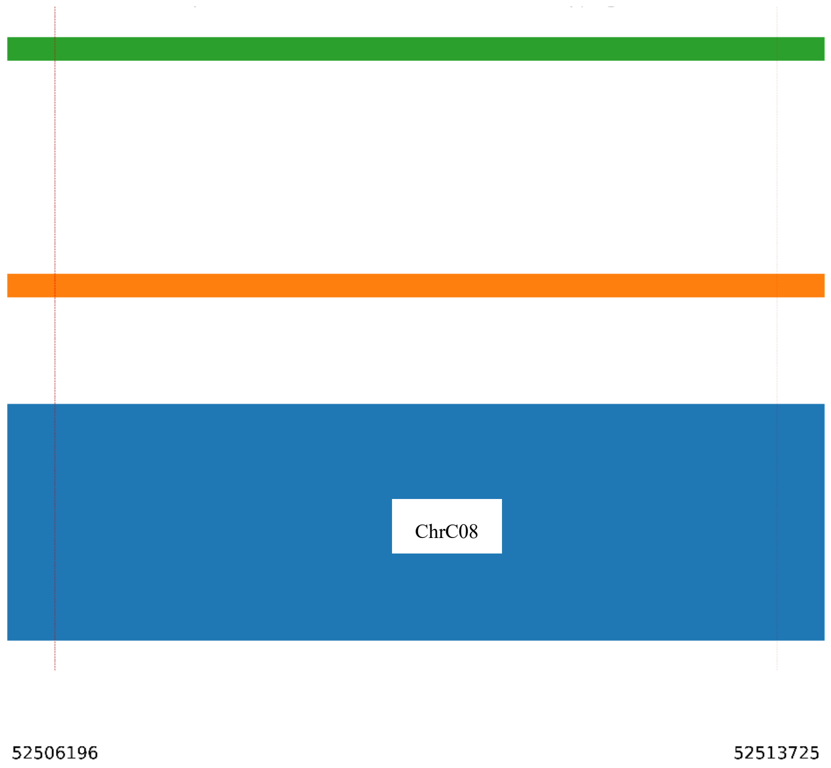

Gap11: Checking the gap regions by Necat

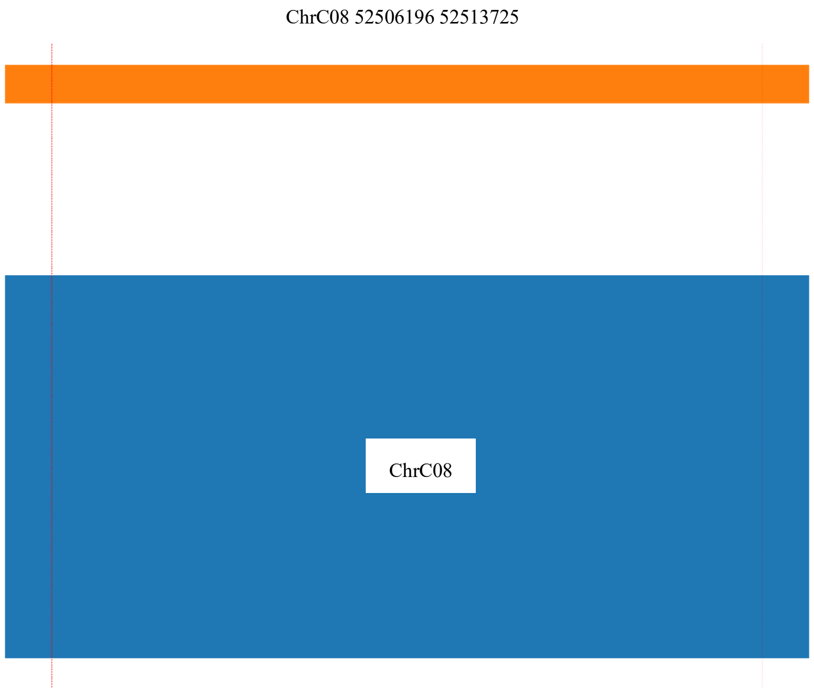

Gap12: Checking the gap regions by HiFi

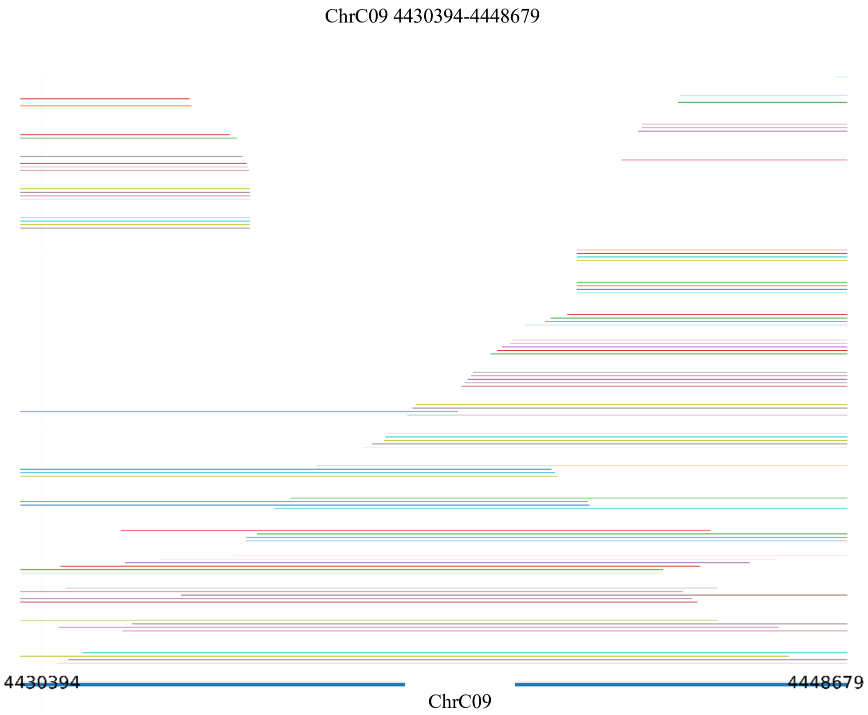

Gap12: Checking the gap regions by Hifiasm

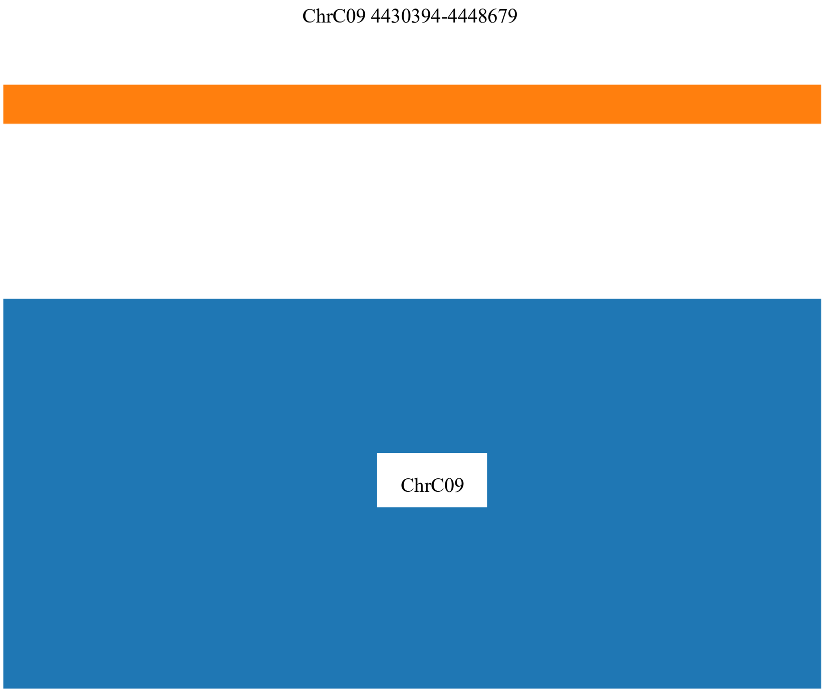

Gap12: Checking the gap regions by Necat

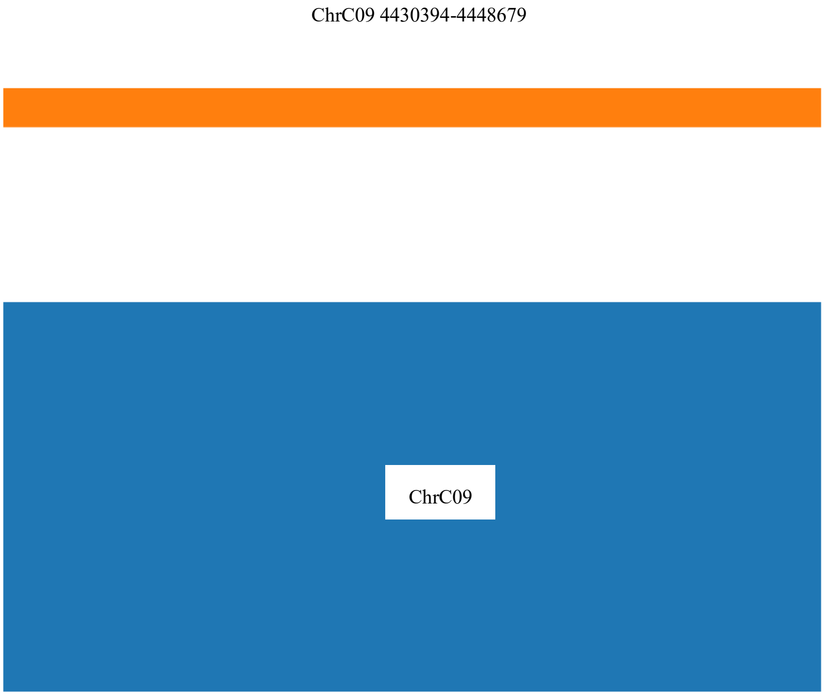

Gap12: Checking the gap regions by ONT

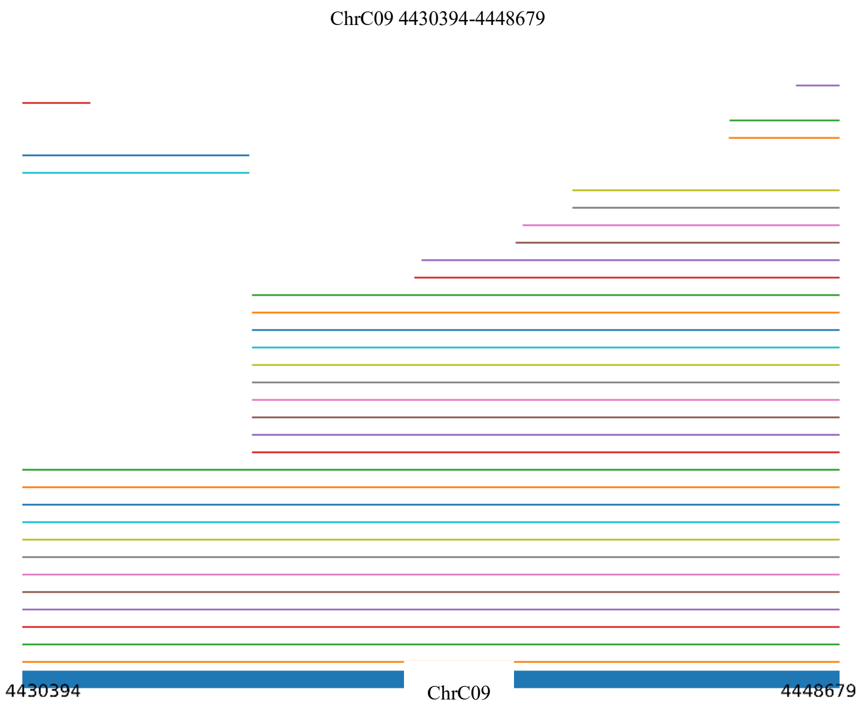

Gap12: Checking the gap regions by Flye

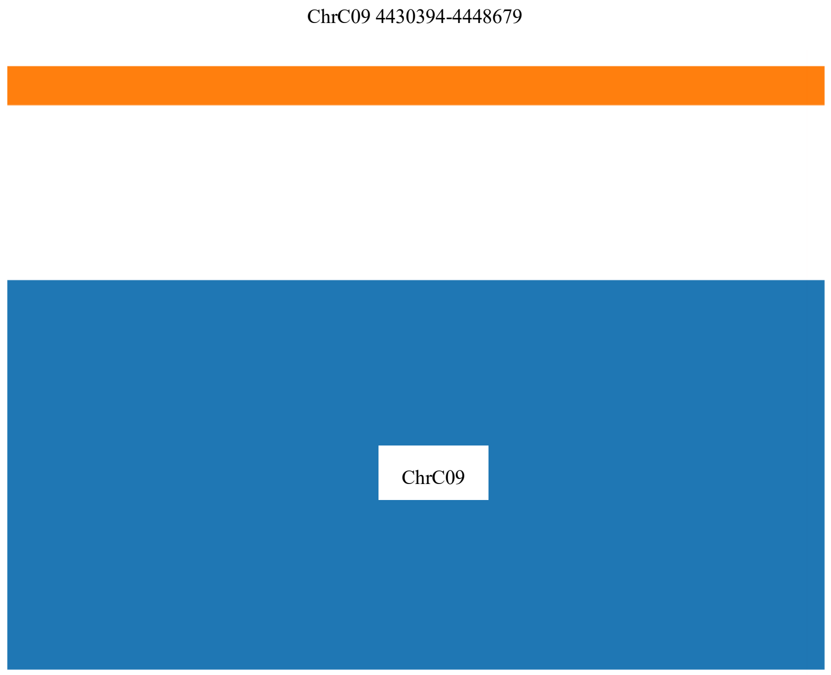

Gap13: Checking the gap regions by HiFi

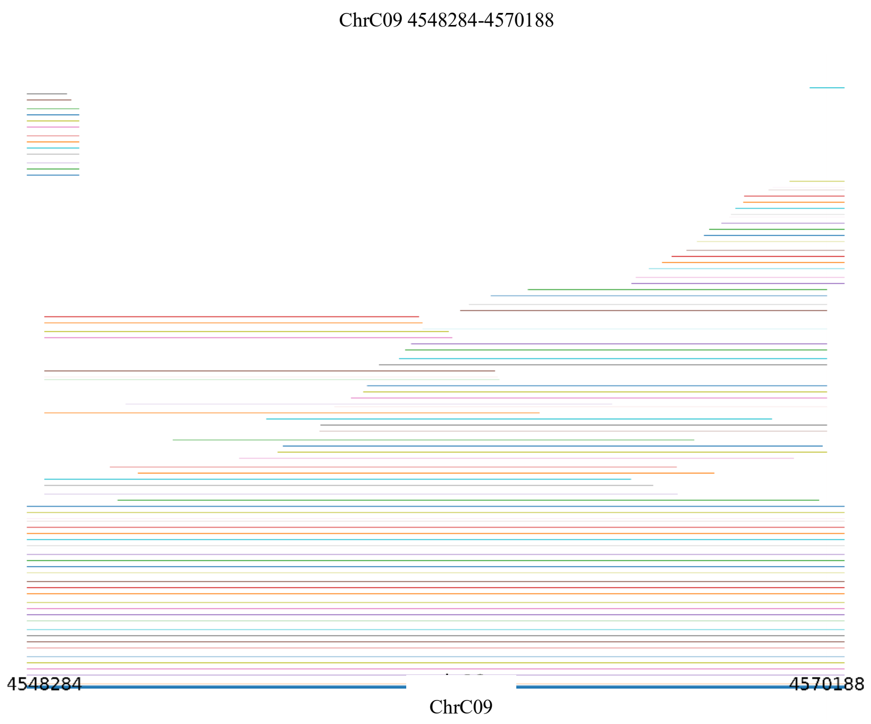

Gap13: Checking the gap regions by Hifiasm

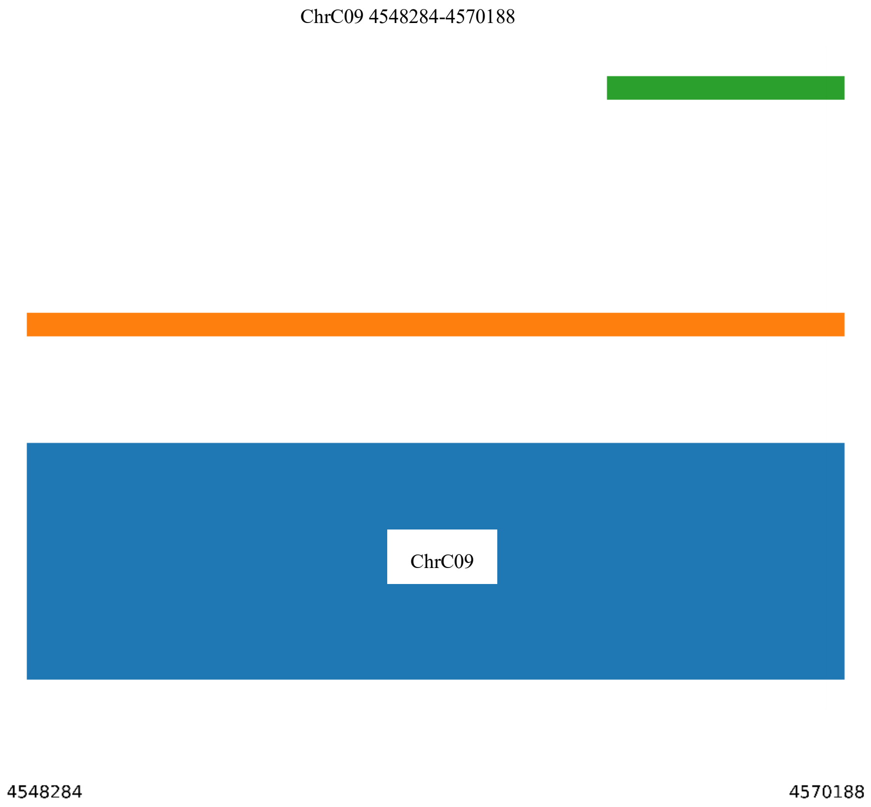

Gap13: Checking the gap regions by Necat

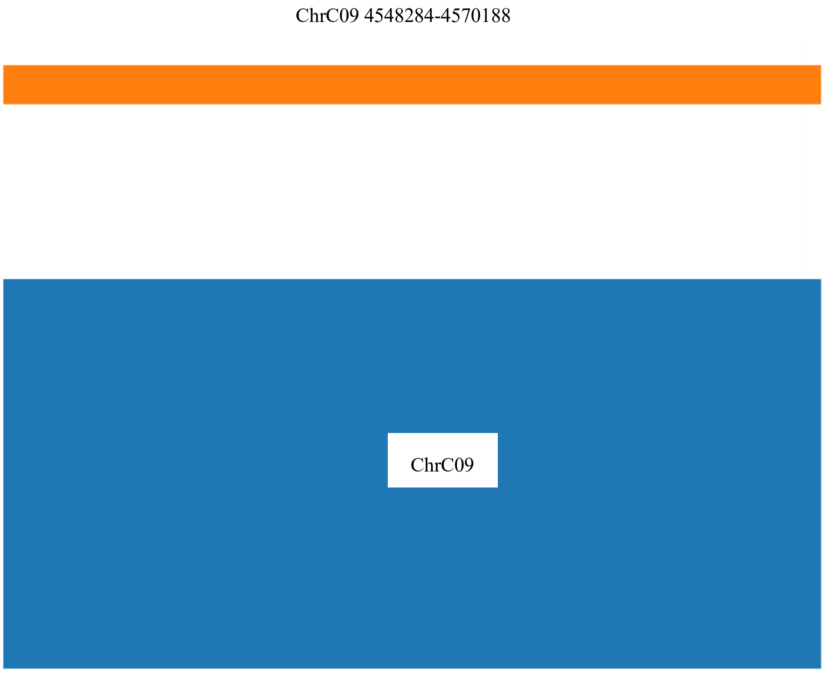

Gap13: Checking the gap regions by ONT

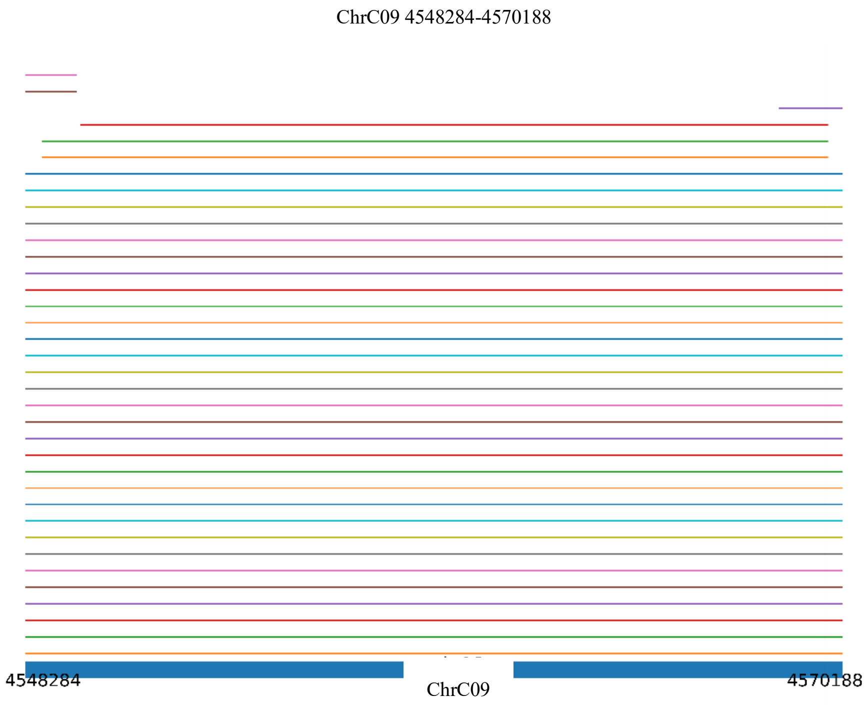

Supplement: Web_Material_uhad171 [file web_material_uhad171.zip › Fig. S2 Gap bridged.pdf]
