# Supplementary figures and images for: A gap-free reference genome reveals structural variations associated with flowering time in rapeseed (Brassica napus)
Source: Hortic Res. 2023 Aug 29;10(10):uhad171. doi: 10.1093/hr/uhad171 (PMC10569240; doi:10.1093/hr/uhad171)

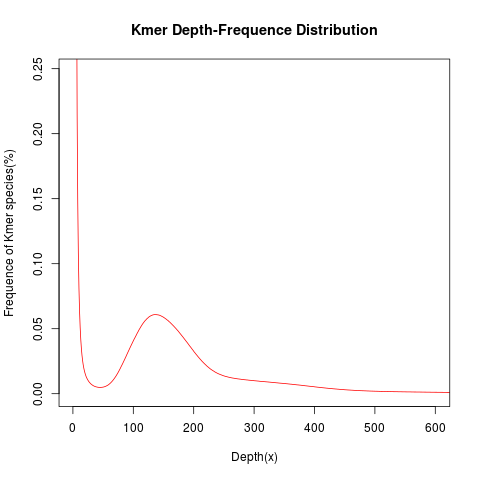

Supplement: Web_Material_uhad171 [file web_material_uhad171.zip › Fig. S1 kmer19.kmer_frequency_species.tif]

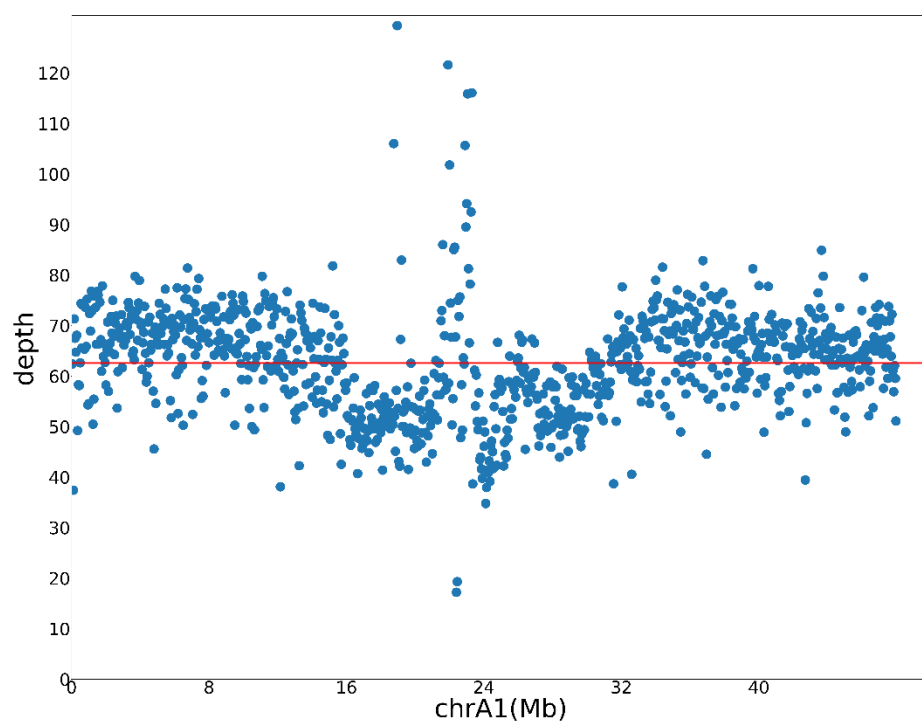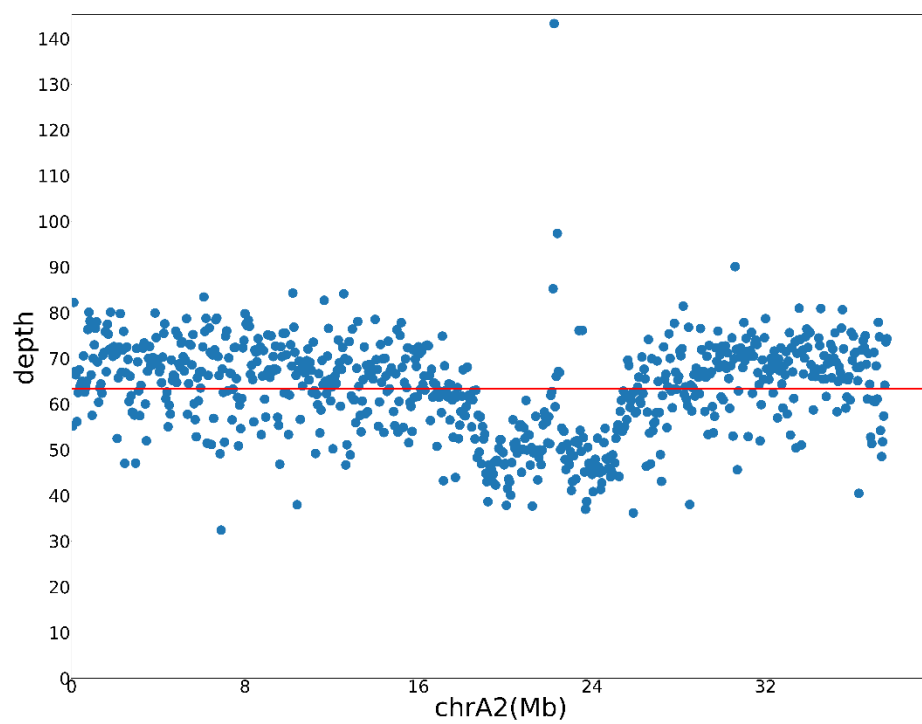

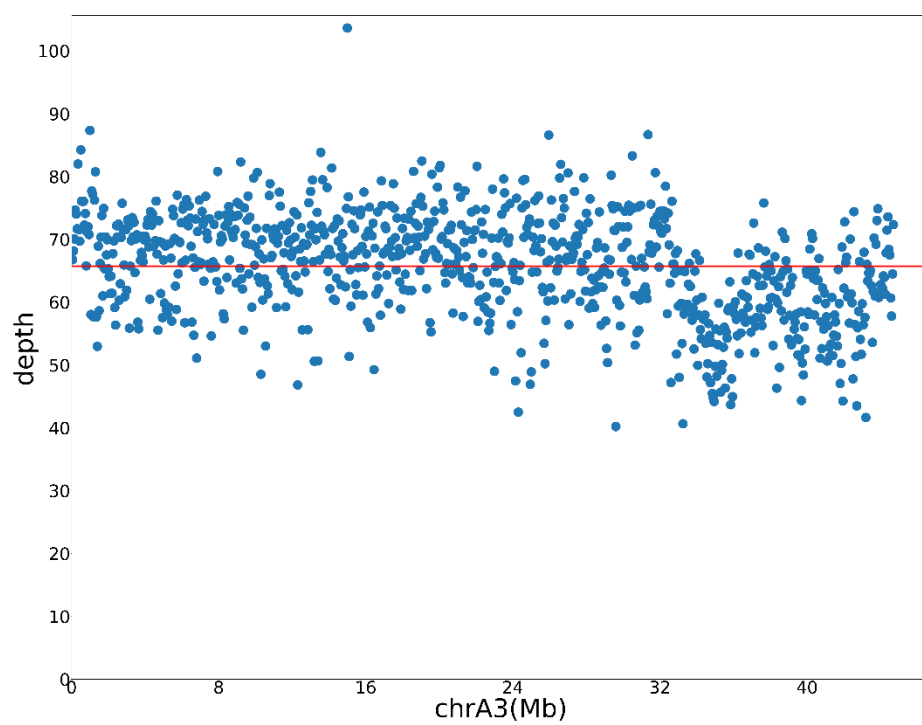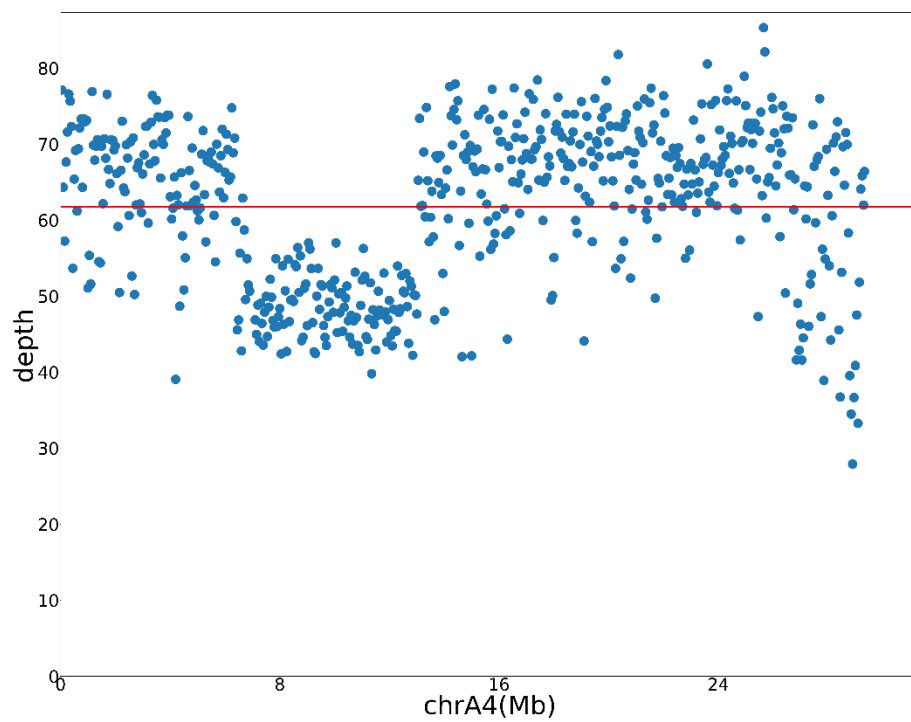

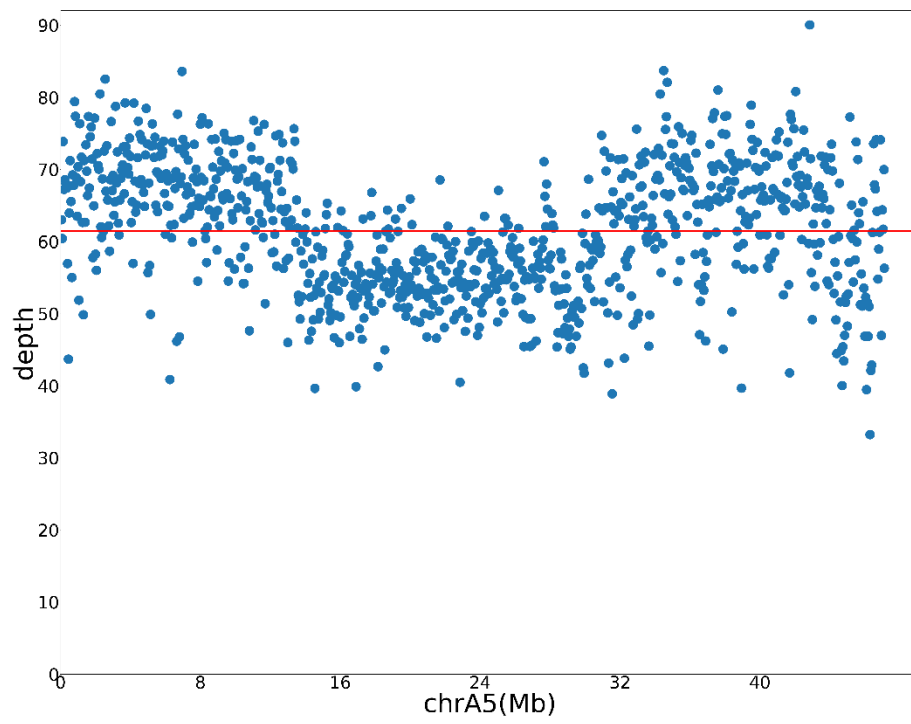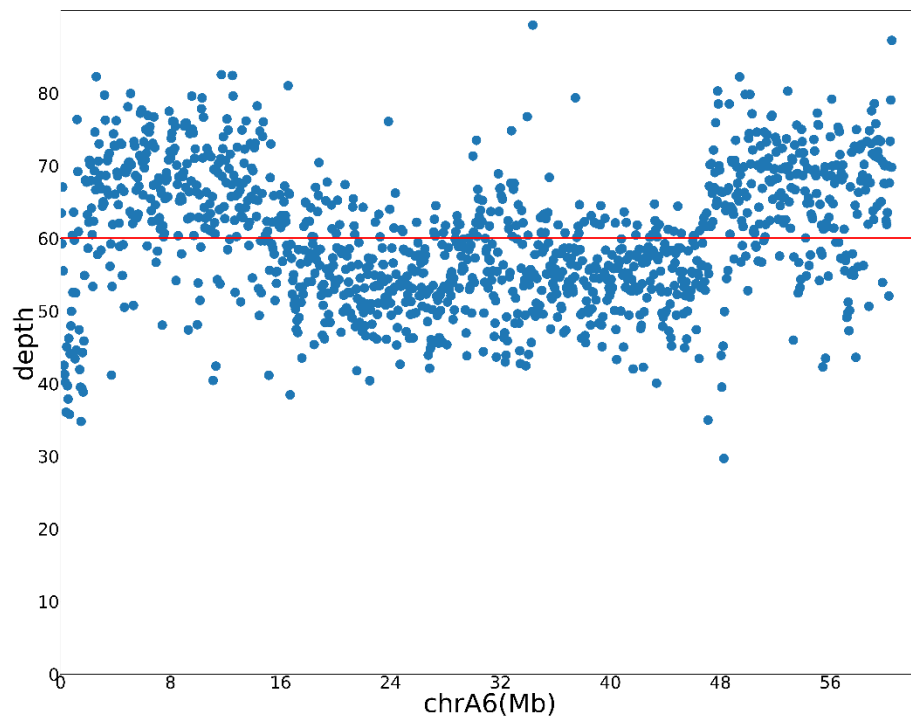

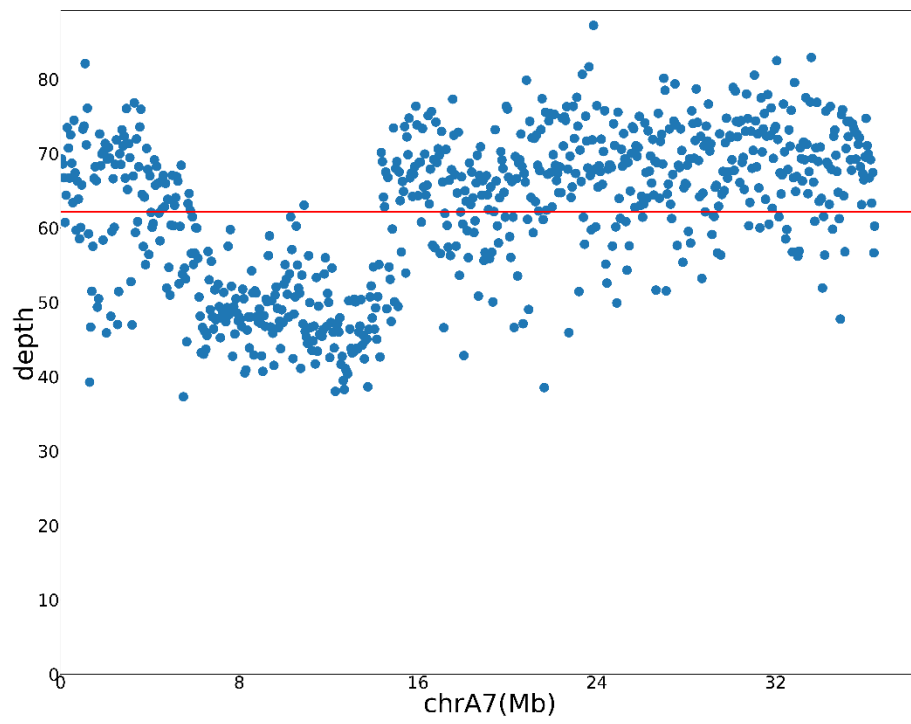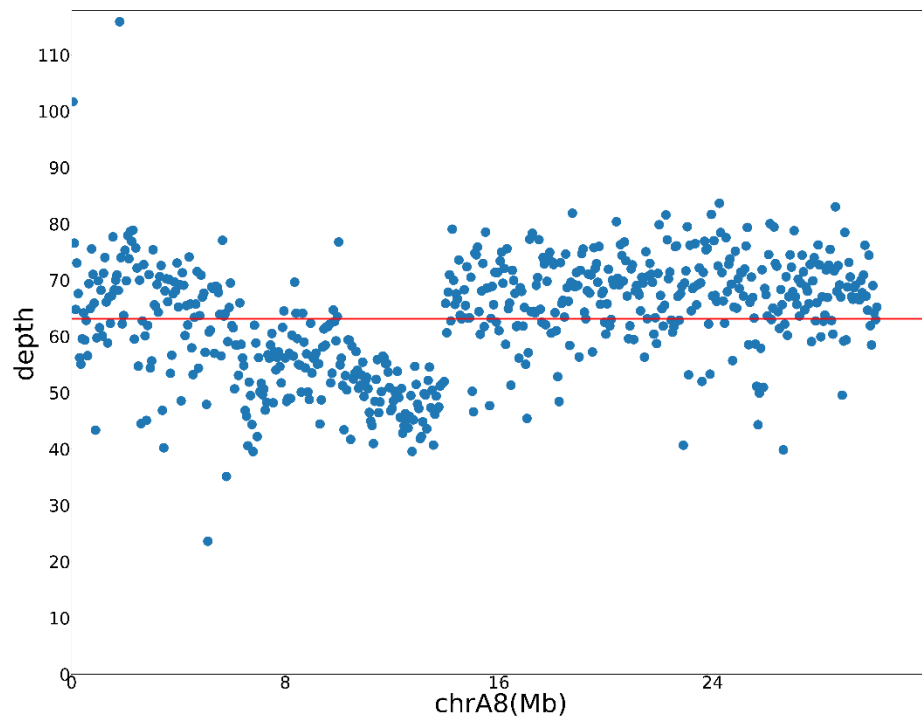

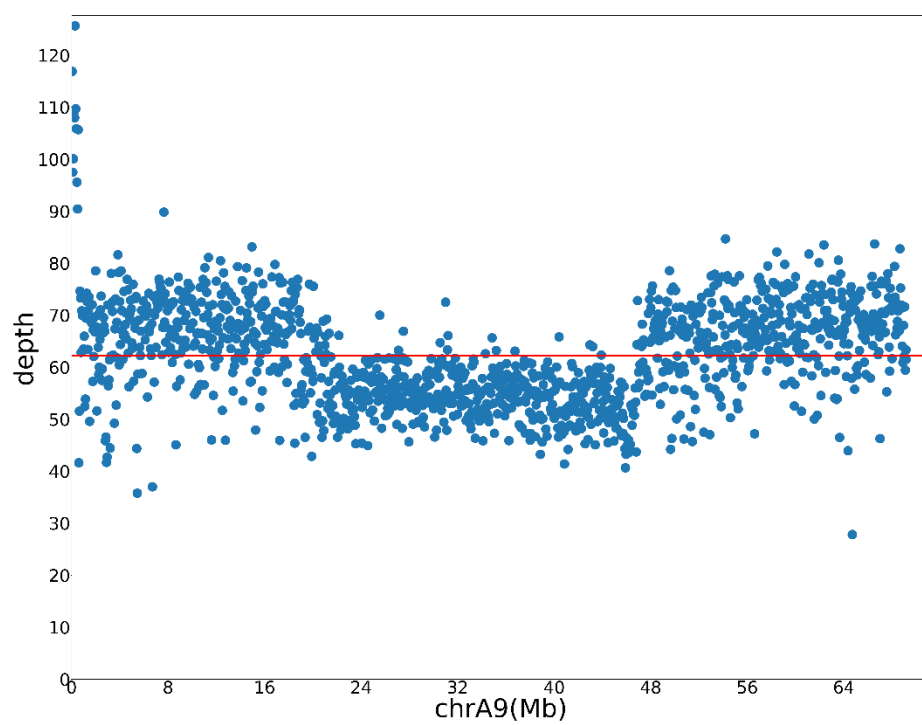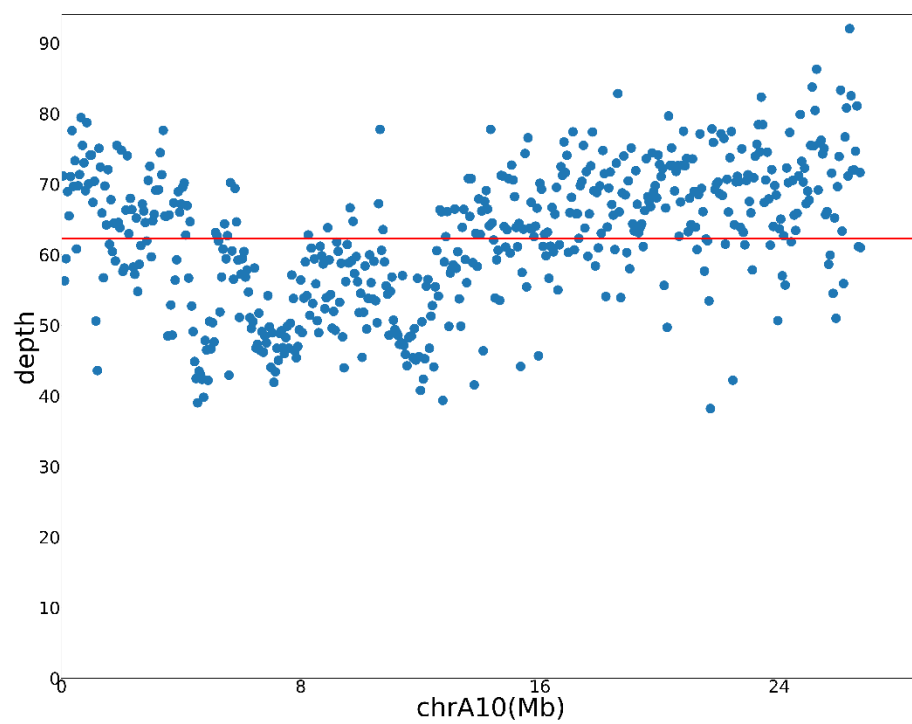

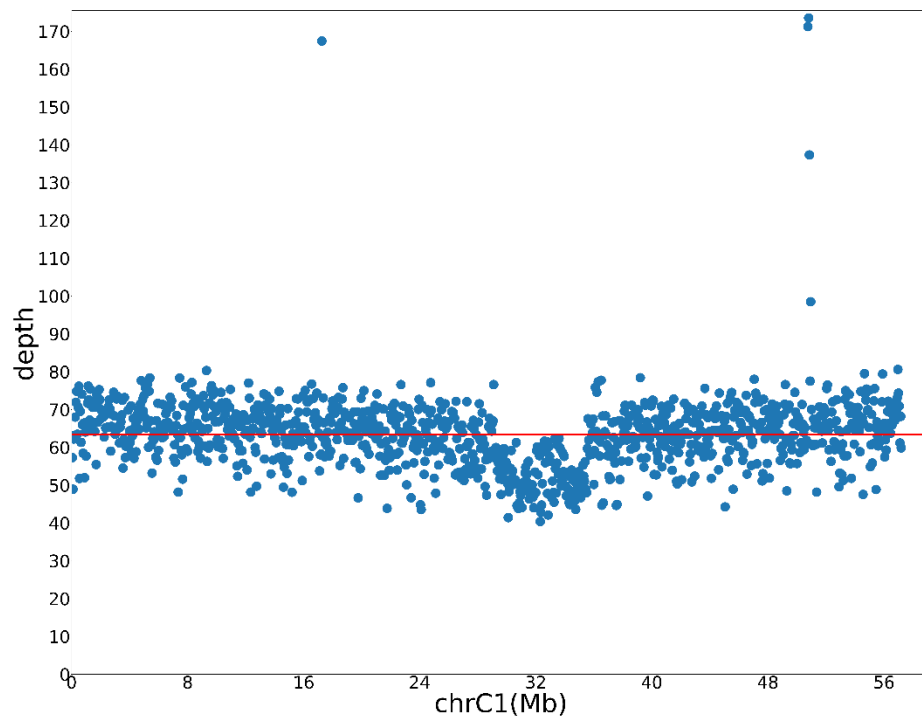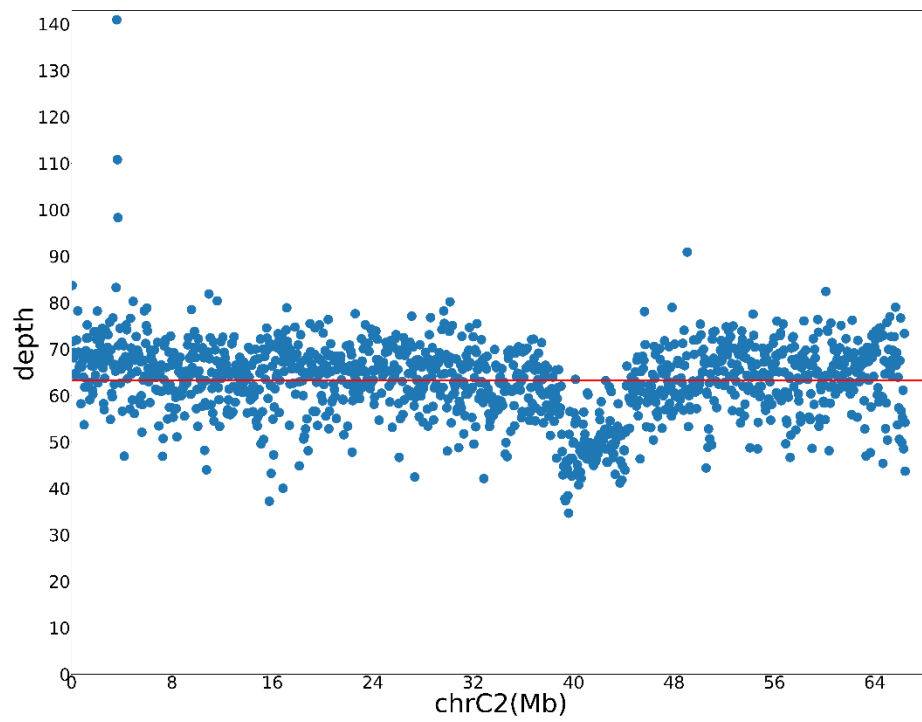

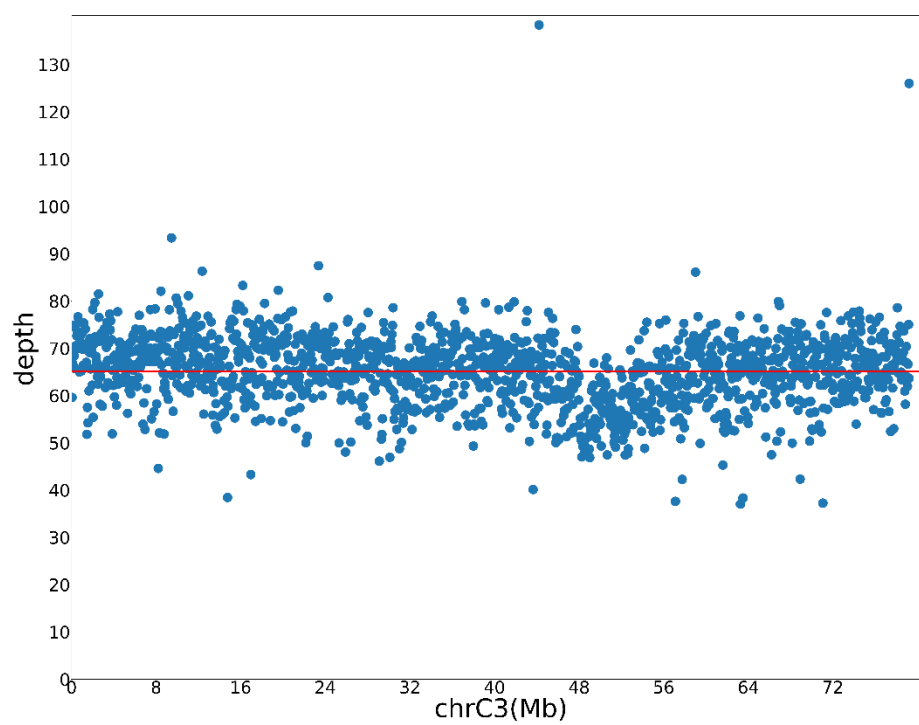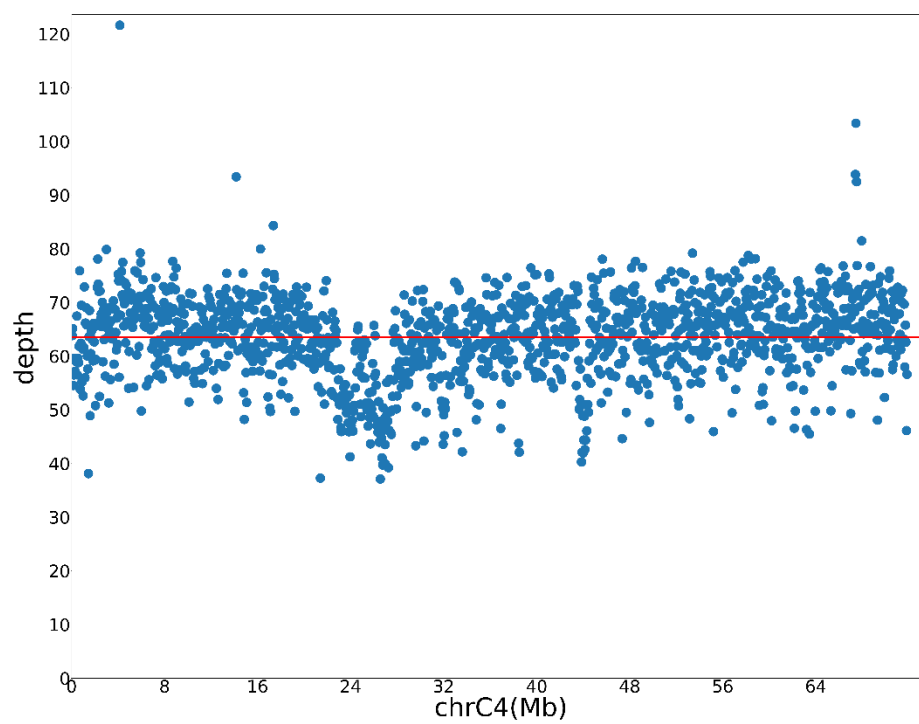

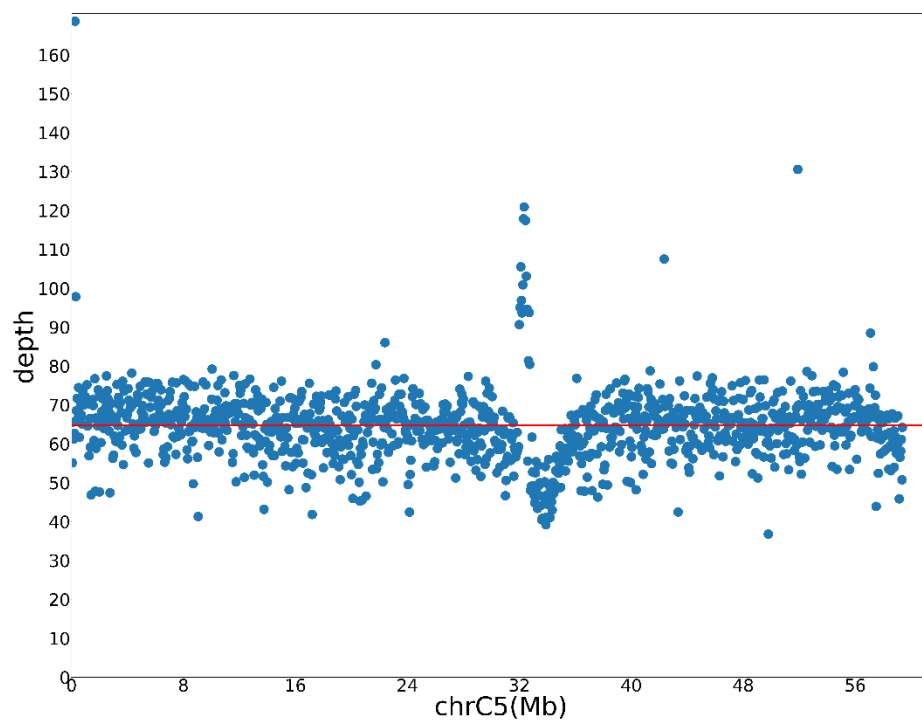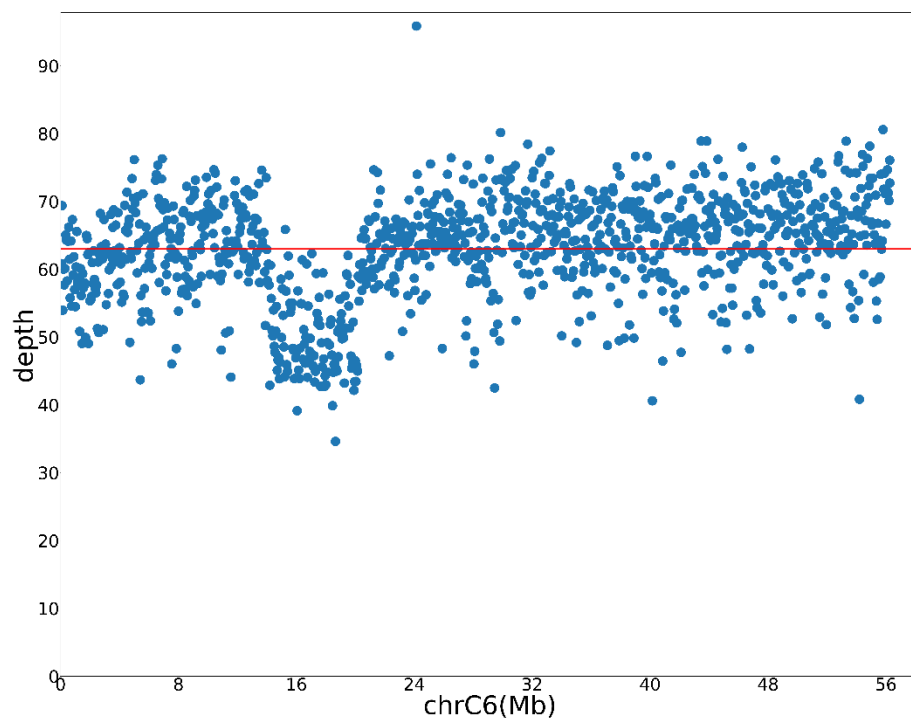

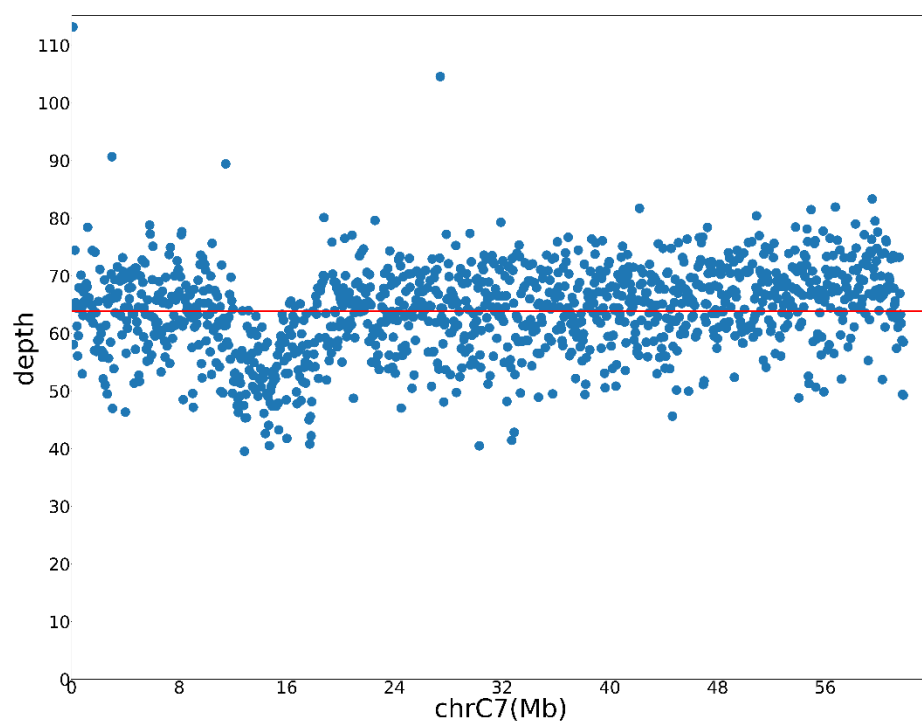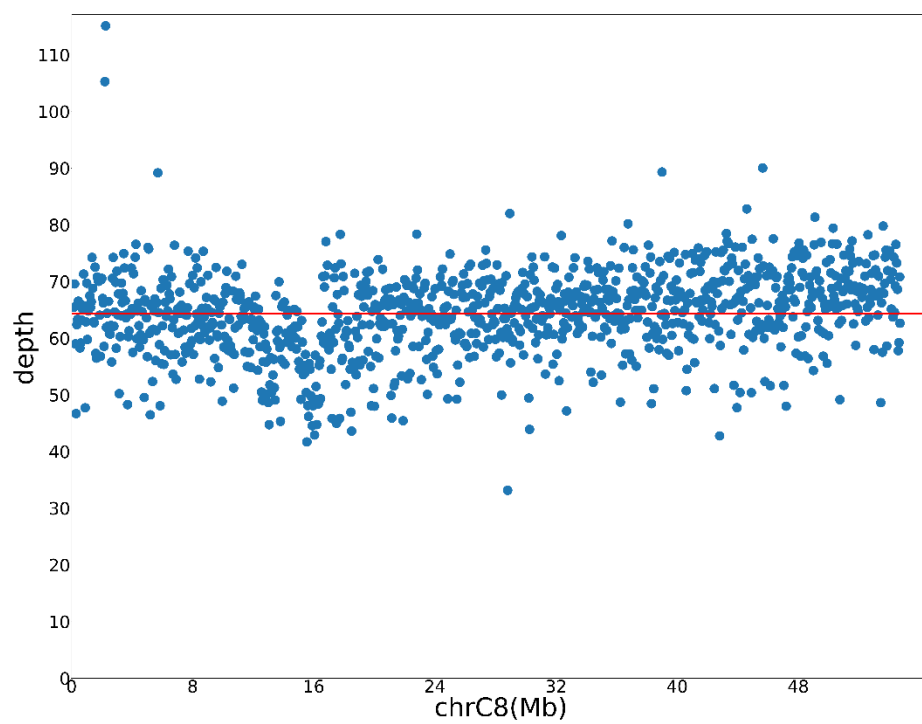

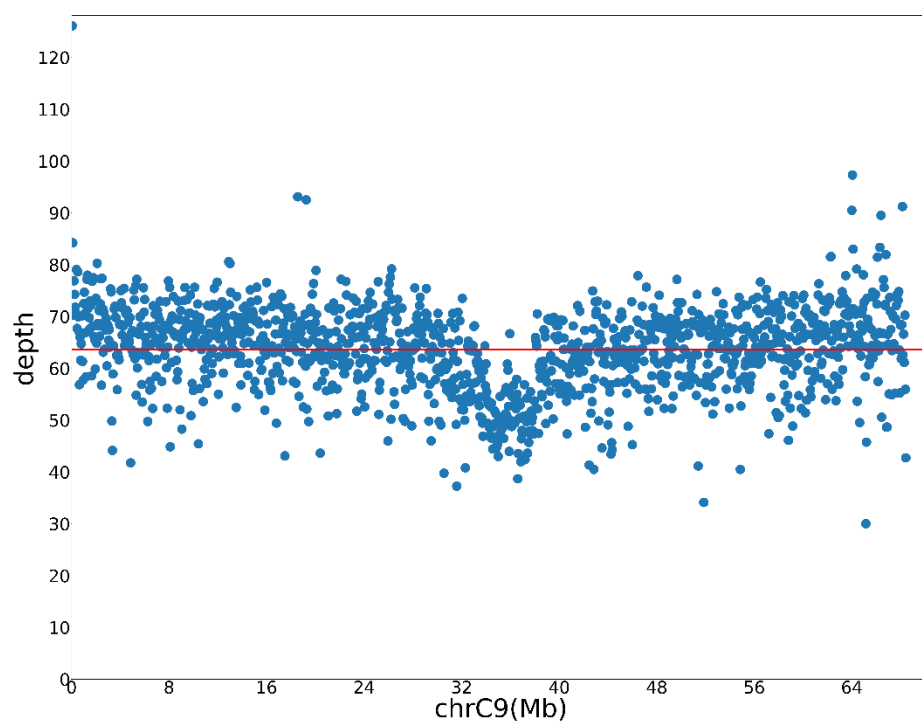

Supplement: Web_Material_uhad171 [file web_material_uhad171.zip › Fig. S3 Depth-HiFi.pdf]

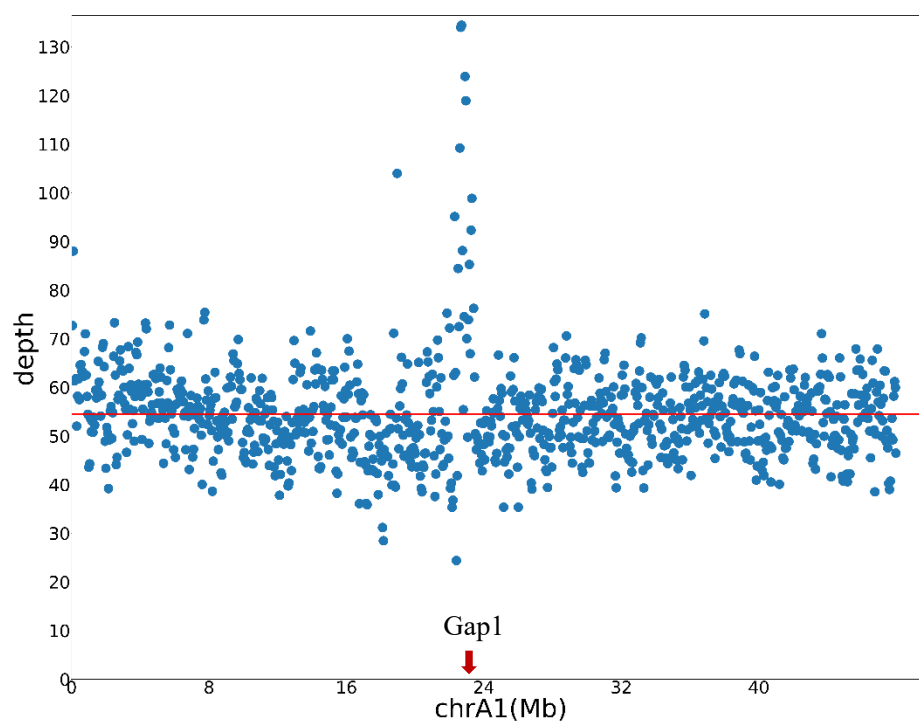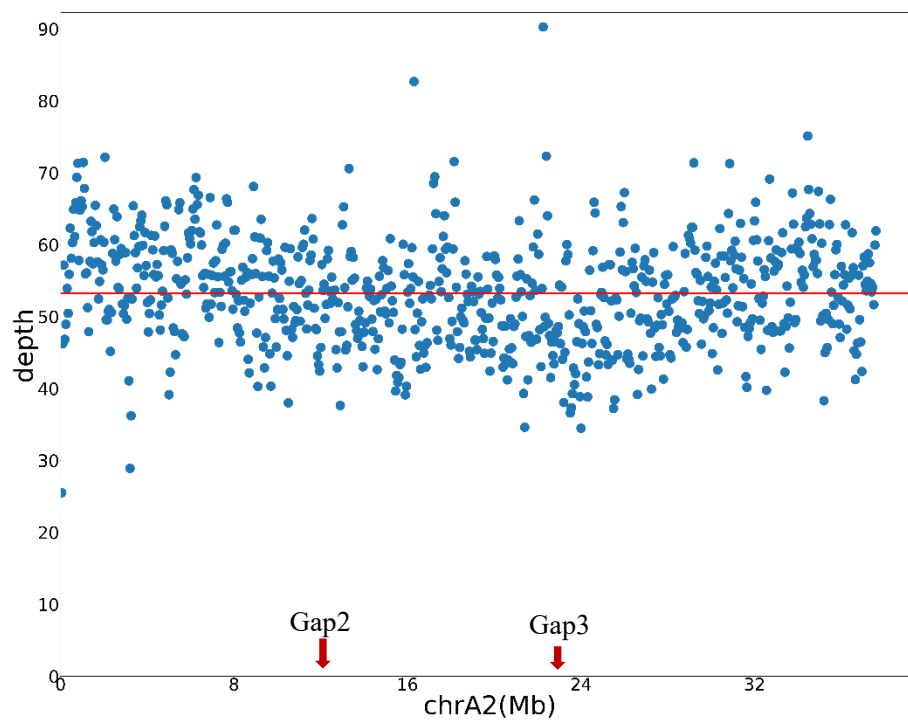

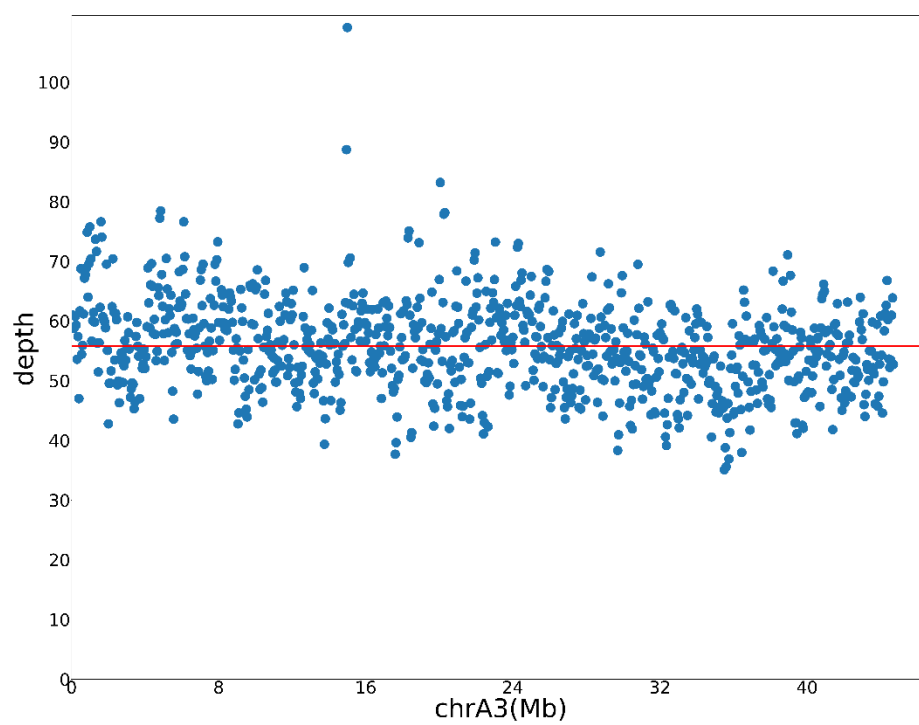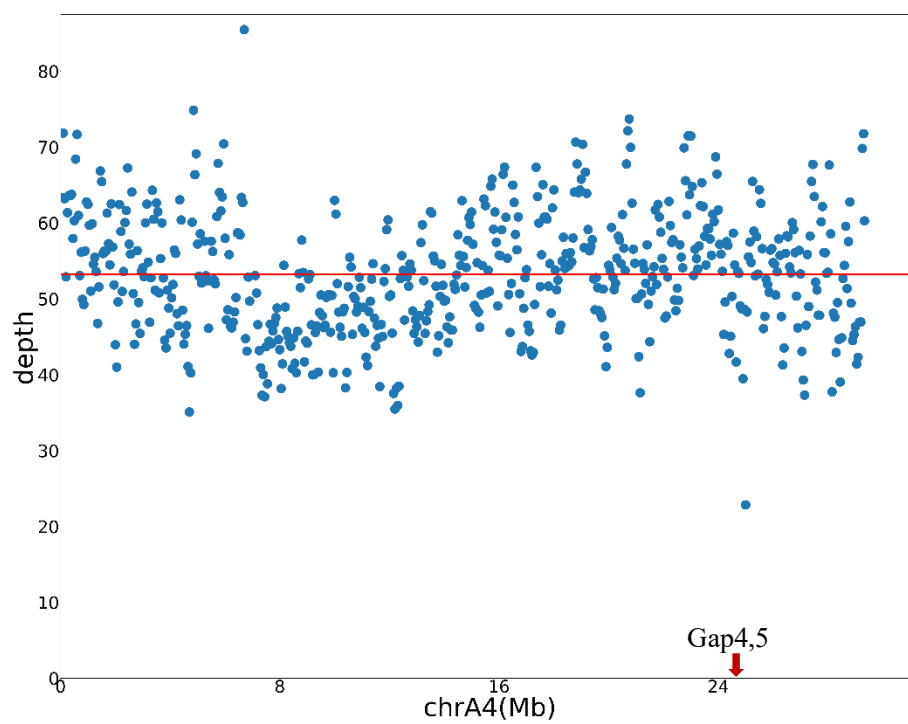

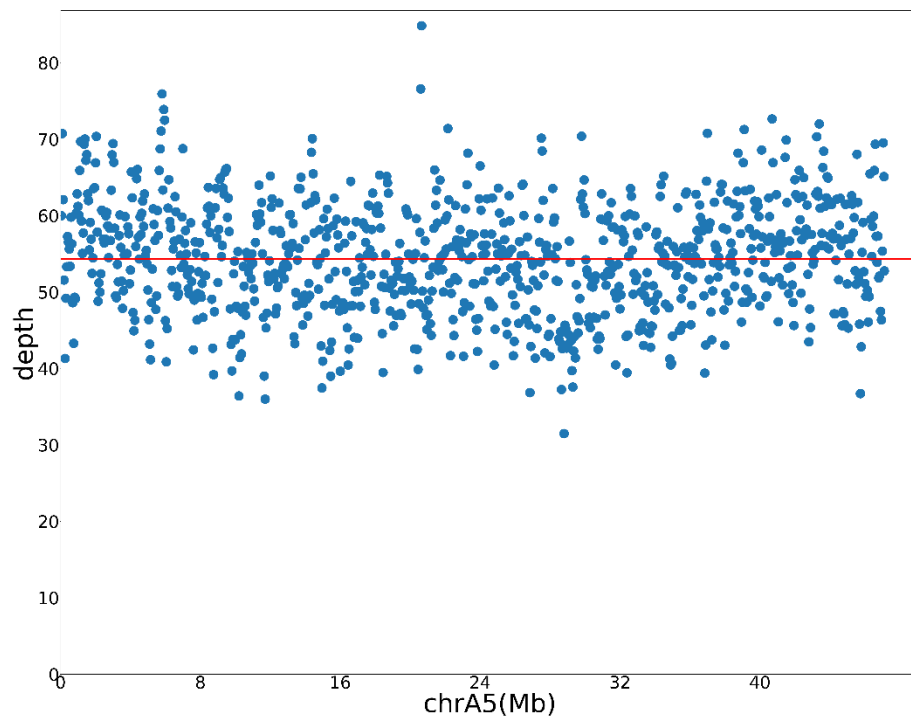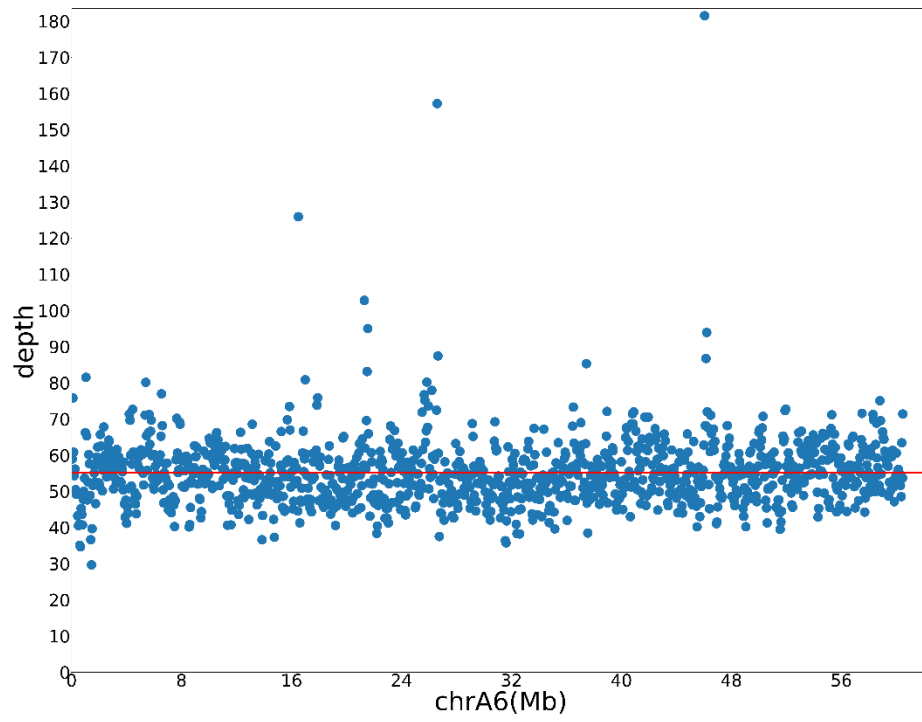

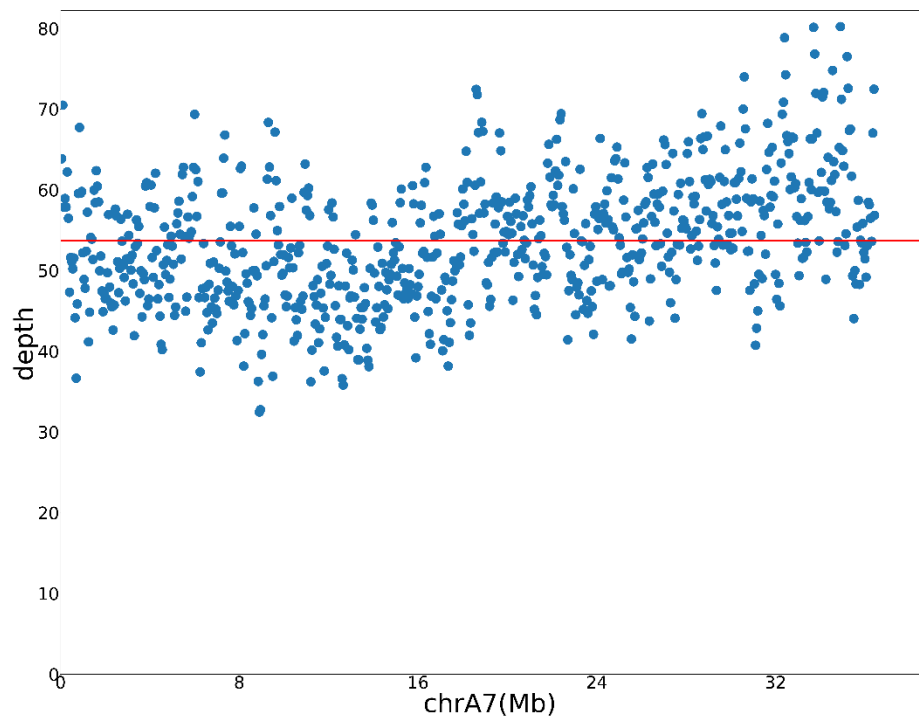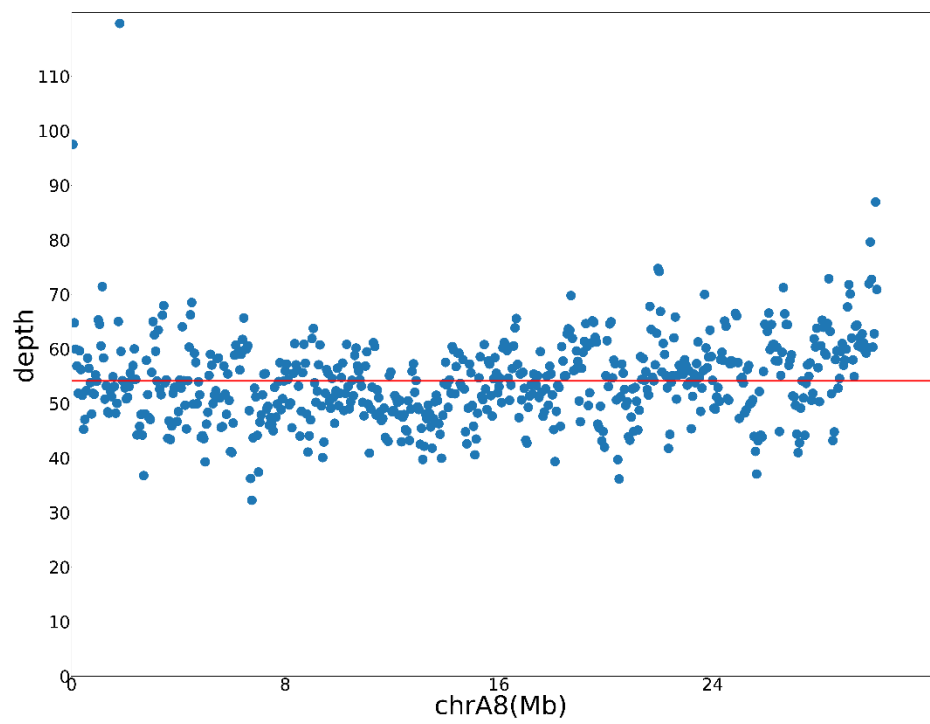

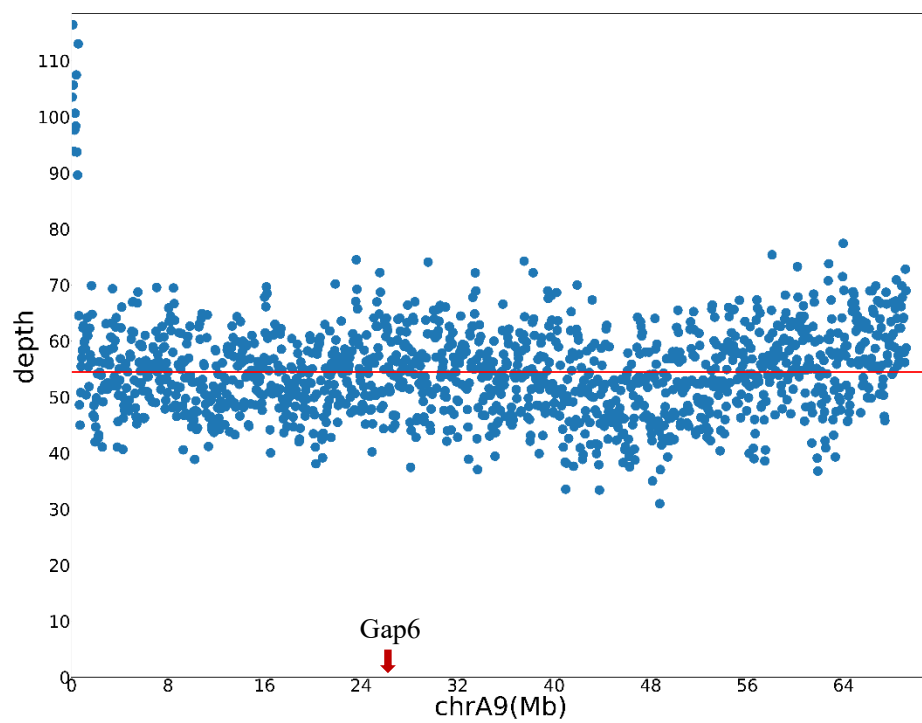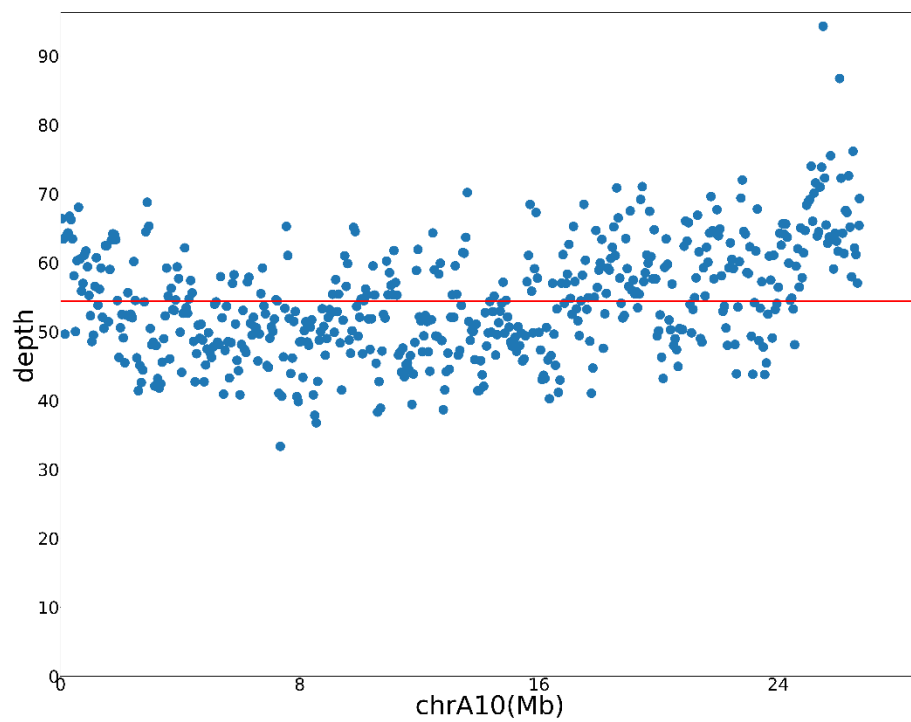

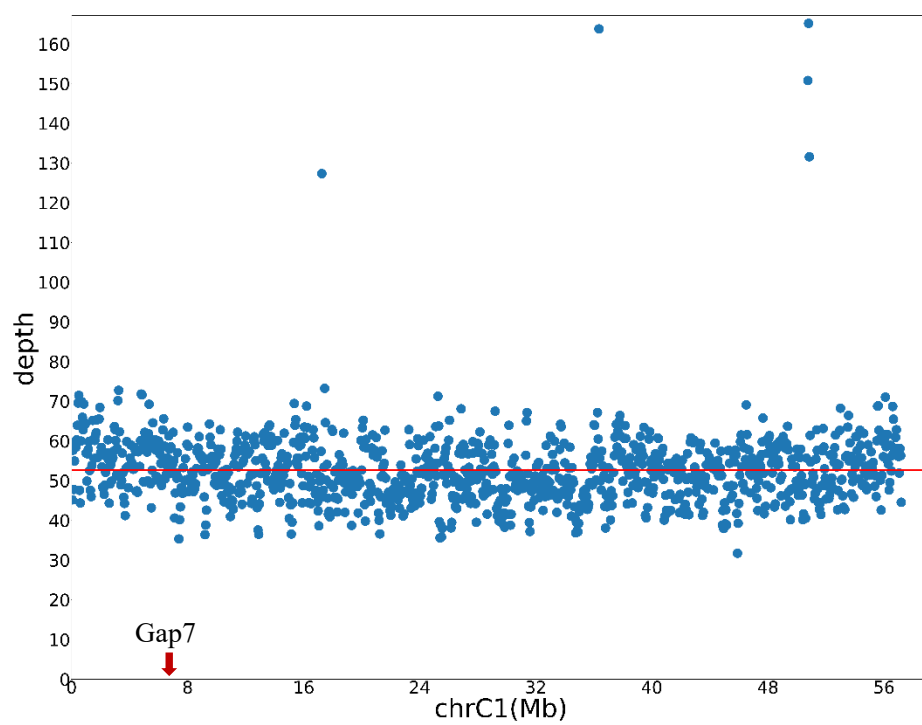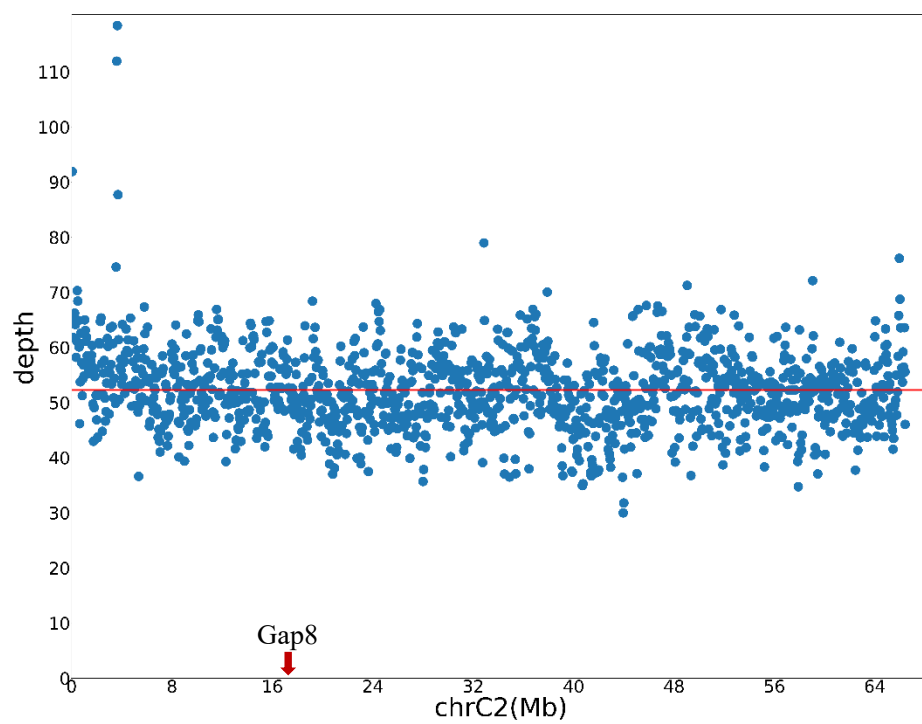

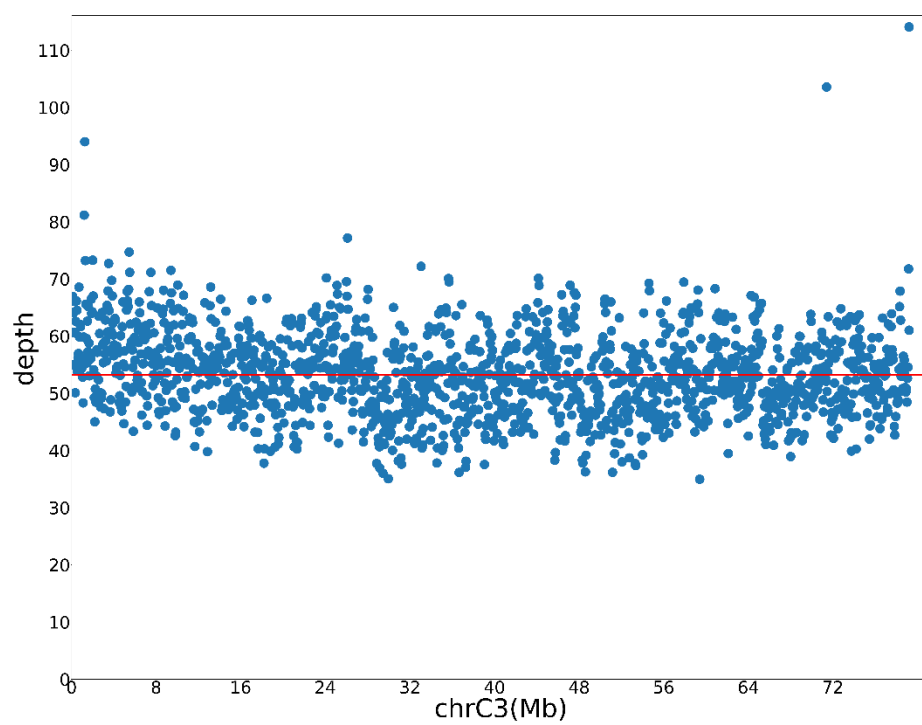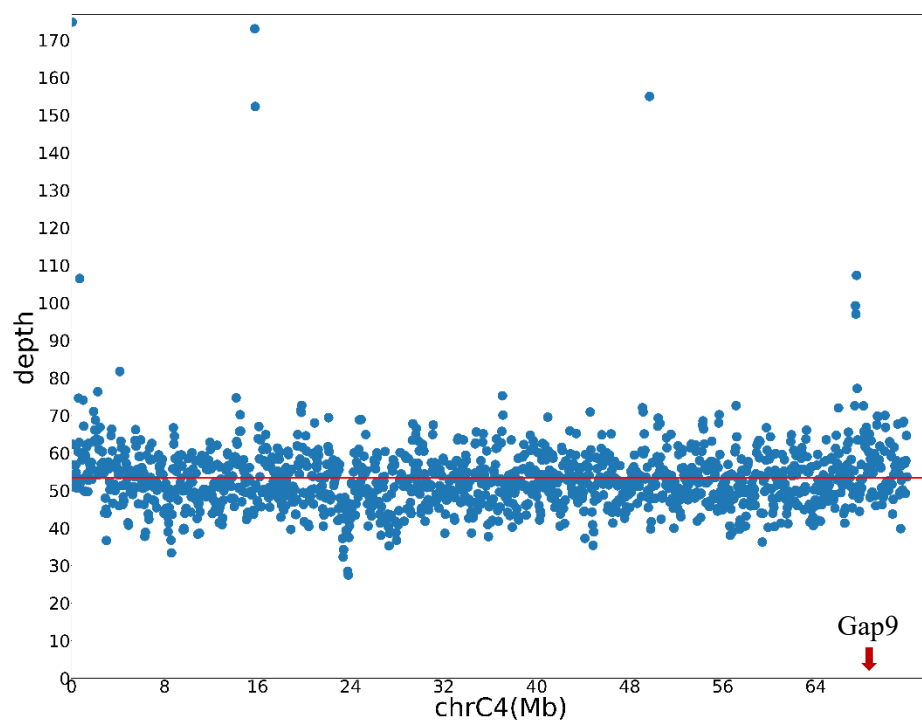

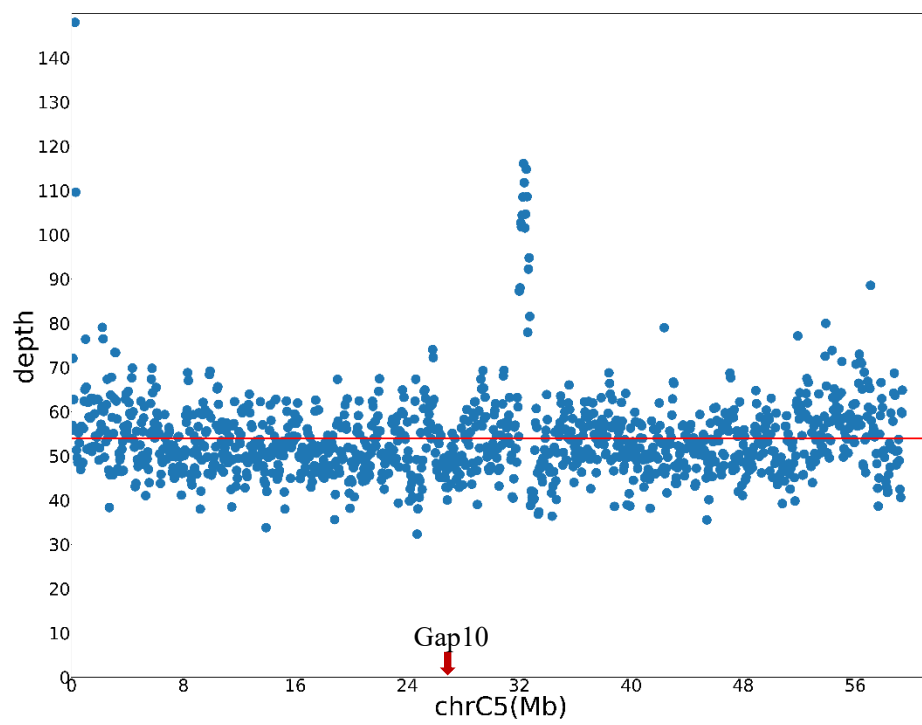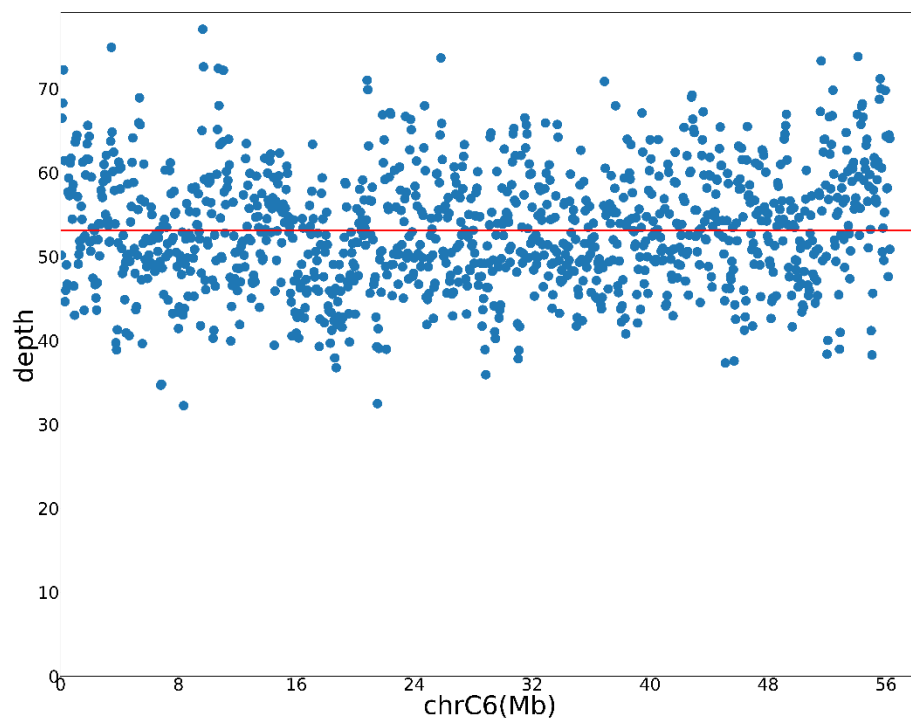

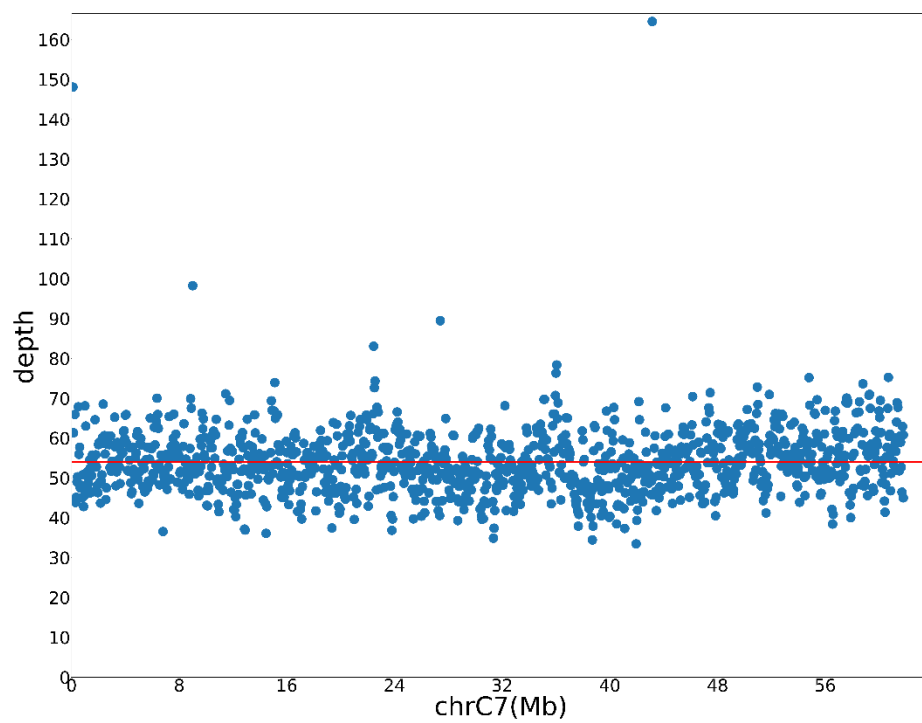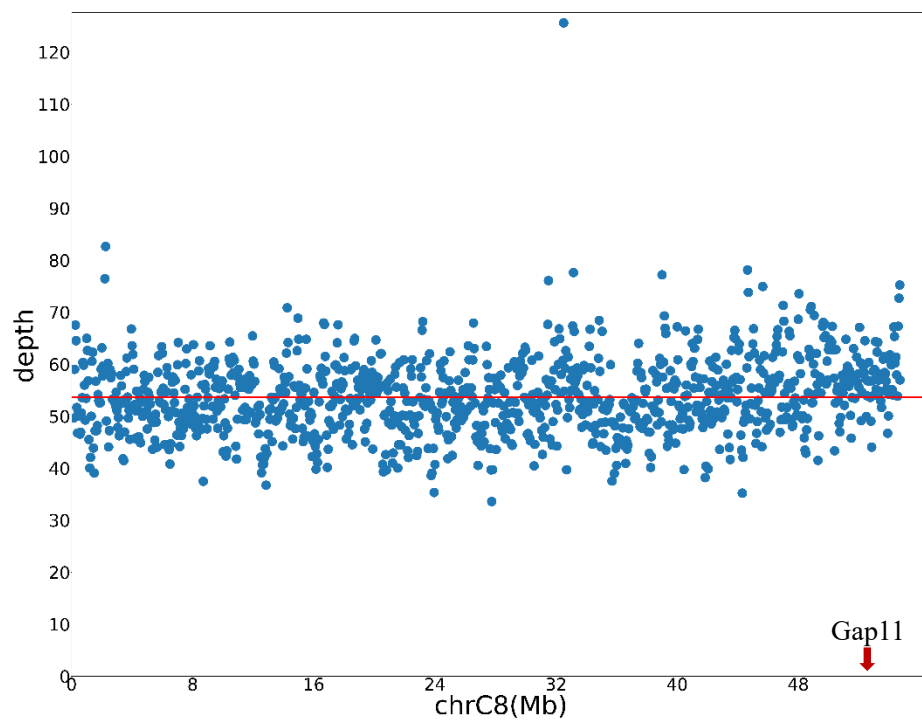

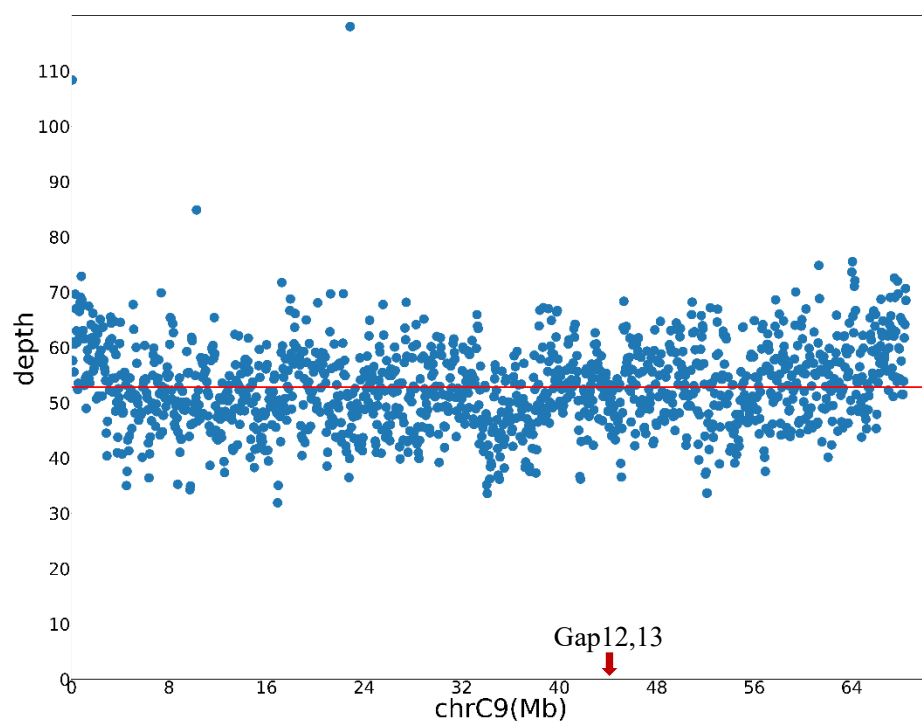

Supplement: Web_Material_uhad171 [file web_material_uhad171.zip › Fig. S4 Depth-ONT.pdf]

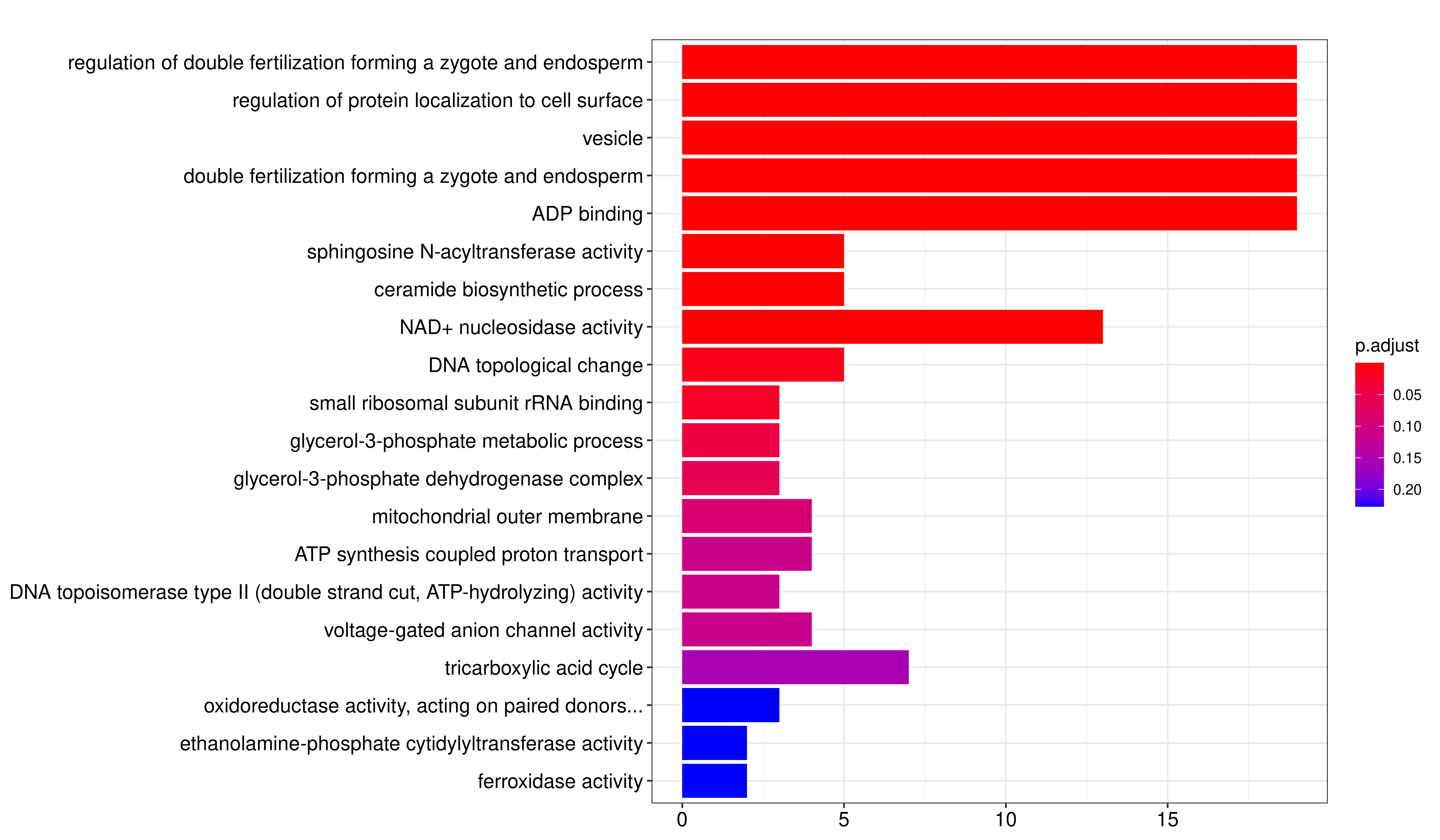

Supplement: Web_Material_uhad171 [file web_material_uhad171.zip › Fig. S5.tif]

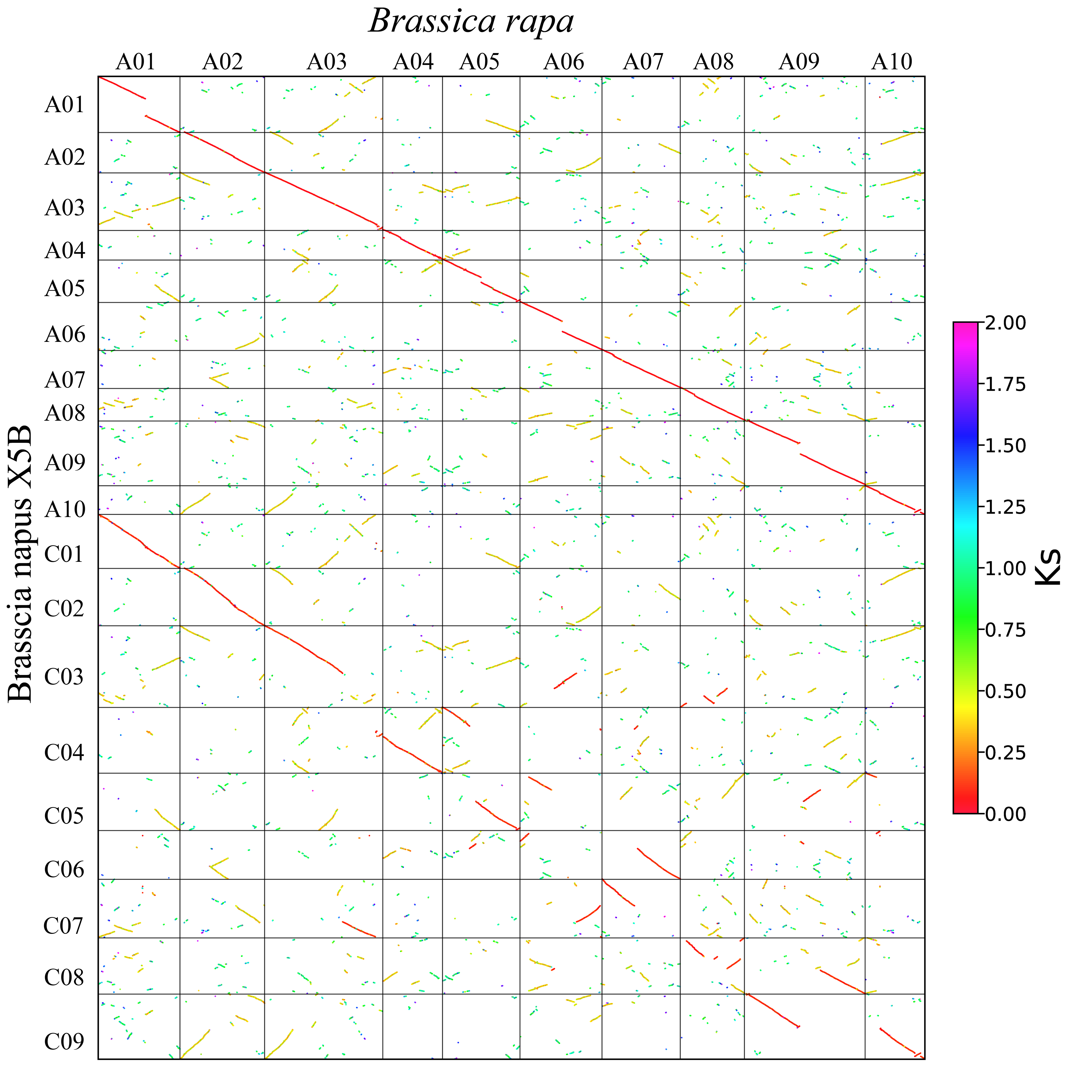

Supplement: Web_Material_uhad171 [file web_material_uhad171.zip › Fig. S6.tif]

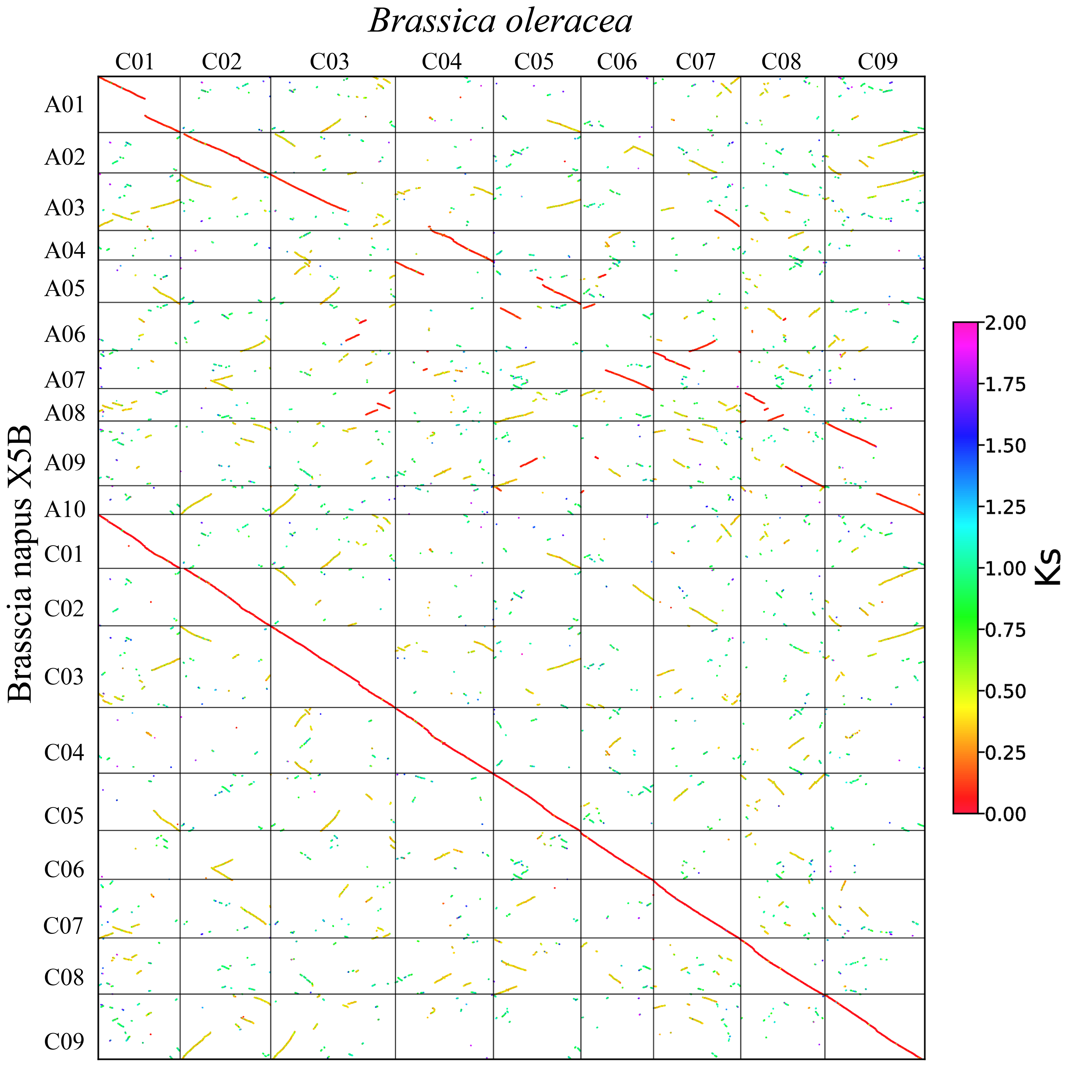

Supplement: Web_Material_uhad171 [file web_material_uhad171.zip › Fig. S7.tif]
